# Supplementary material for: Computing high-degree polynomial gradients in memory
Source: Nat Commun. 2024 Sep 18;15:8211. doi: 10.1038/s41467-024-52488-y (PMC11411077; doi:10.1038/s41467-024-52488-y)
Supplement: Supplementary file 1 — Supplementary Information [file 41467_2024_52488_MOESM1_ESM.pdf]

Computing High-Degree Polynomial Gradients in Memory

T. Bhattacharya[1*], G. Hutchinson[1], G. Pedretti[2], X. Sheng,[2] J. Ignowski[2], T. Van Vaerenbergh[3], R. Beausoleil[3], J.P. Strachan[4] & D.B. Strukov[1*]

Supplementary Information

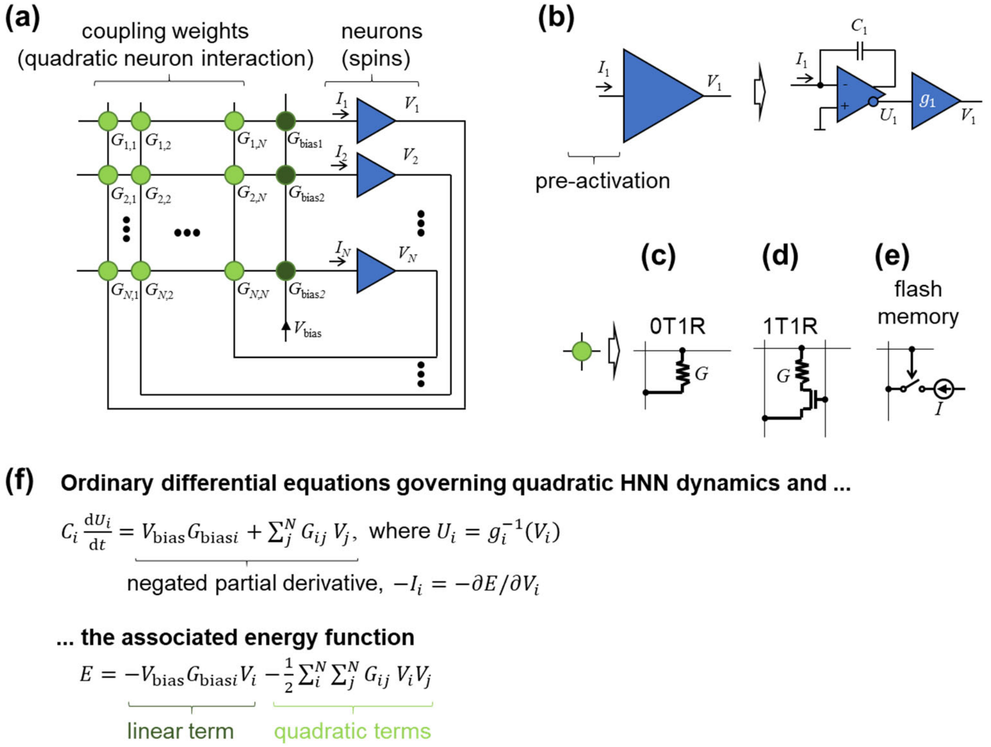

Figure S1. Quadratic Hopfield neural network: (a) Neural network structure, (b) examples of neuron and (c-e) synapse implementations with (c,d) memristive and (e) flash memory device technologies, and (f) equations describing network operation. A shown neuron implementation is based on a differential-output operational amplifier (opamp) with capacitive feedback, whose noninverting output is connected to the second opamp implementing nonlinear activation, e.g., sigmoid function. Due to virtual ground configuration, the current to the first opamp is effectively neuron pre-activation, i.e., $I_i = \sum G_{ij} V_j + G_{\text{bias}i} V_{\text{bias}}$. In panel c, “0T1R” stands for zero transistors plus one resistive switching element (memristor) per memory cell, while in panel d, “1T1R” stands for one transistor plus one memristor. Note that the figure shows HNN with type I dynamics according to classification in Ref. 16, i.e., does not include $-U_i$ “leakage” term in the right-hand side of differential equations that are common to a more widespread HNN implementation with dynamics type II. Also, in principle, type I dynamics implies unbounded values of U, while they would be bounded in the practical hardware implementation. This introduces a nonlinear “squishing” function for U that is, for simplicity, not shown in panel f equations. However, such an additional squishing function does not modify the minima of the original energy function.

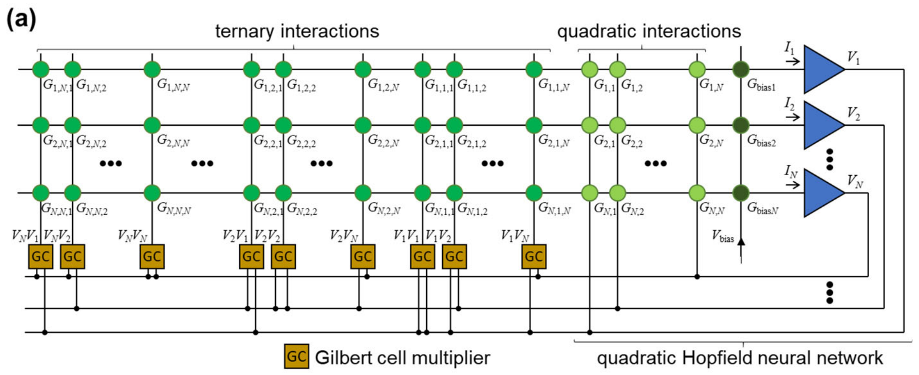

(b) Ordinary differential equations governing 3-rd order HNN dynamics and ...

$$C_i \frac{\mathrm{d}U_i}{\mathrm{d}t} = V_{\text{bias}} G_{\text{bias}i} + \sum_j^N G_{ij} V_j + \sum_j^N \sum_k^N G_{ijk} V_j V_k, \text{ where } U_i = g_i^{-1}(V_i)$$

negated partial derivative, $-I_i = -\partial E / \partial V_i$

... and the associated energy function

$$E = -V_{\text{bias}} G_{\text{bias}i} V_i - \frac{1}{2} \sum_i^N \sum_j^N G_{ij} V_i V_j - \frac{1}{3} \sum_i^N \sum_j^N \sum_k^N G_{ijk} V_i V_j V_k$$

linear term — quadratic terms — ternary terms

Figure S2. High-order Hopfield neural network: (a) The network structure and (b) equations governing operation. For clarity, the figure shows a third-order HNN (of type I, similar to Figure S1) with a regular implementation at the cost of some redundancy, which is chosen to highlight how such third-order (K=3) HNN can be extended to higher-order (K>3) networks. Specifically, the shown implementation of third-order interactions employs N^2 columns and hence N^3 synapses. Because of the symmetric coupling matrix and typically employed multi-linear energy functions, the number of columns can be reduced to (N-1)N/2, while the number of synapses to (N-1)N^2/2 in a more optimal implementation for third-order HNNs.

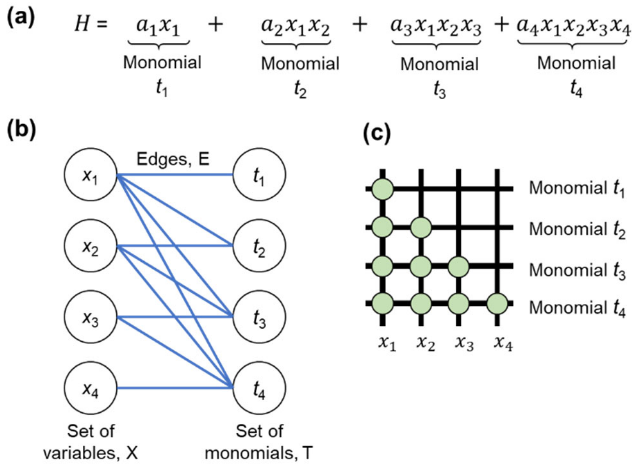

Figure S3. Mapping polynomial to the crossbar array: (a) Considered multi-linear polynomial example, (b) its graph representation, and (c) corresponding crossbar circuit implementation.

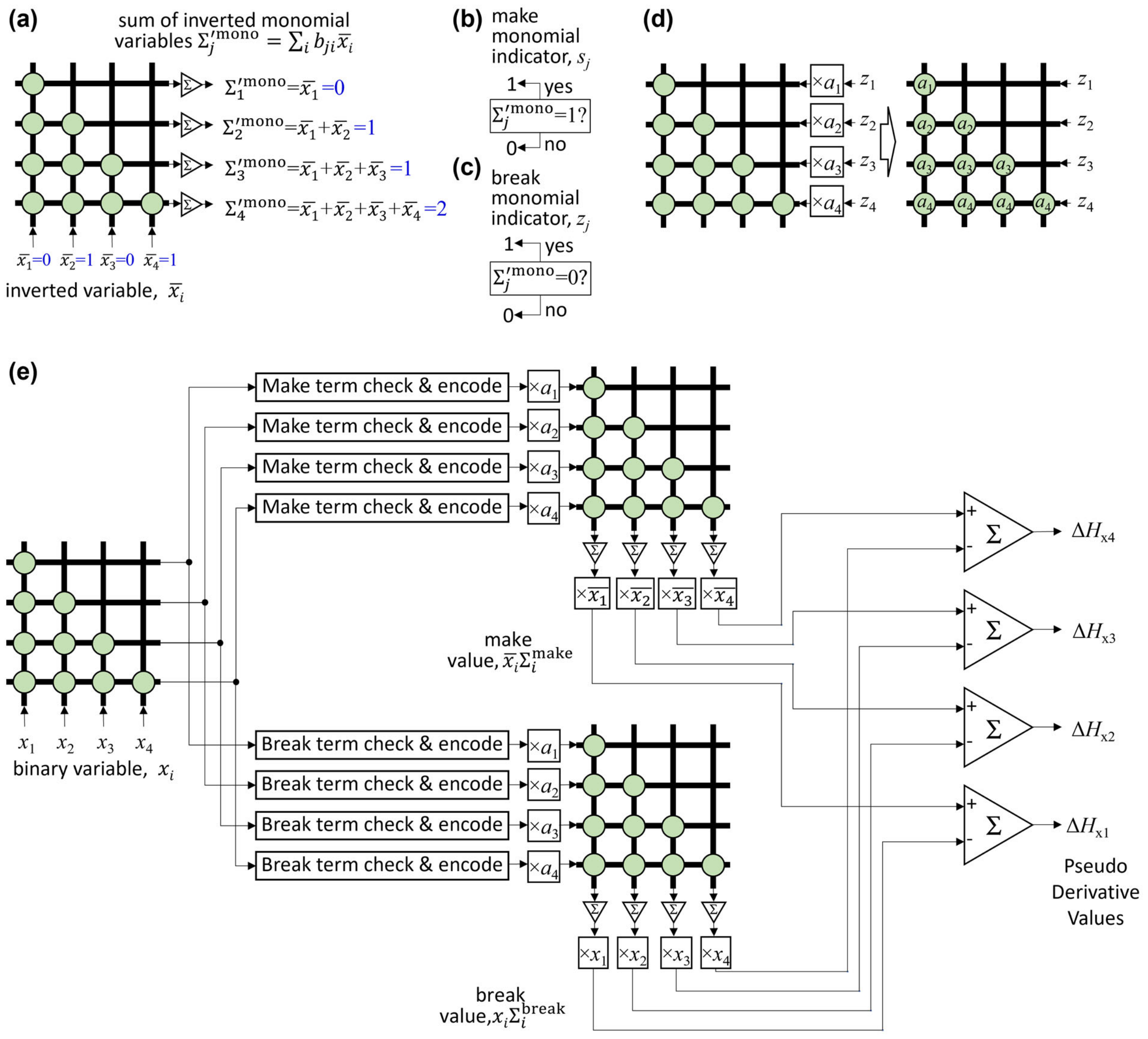

Figure S4. Simplified periphery and high-throughput extensions: (a-d) Simplifying peripheral circuitry by (a) inverting variable inputs in the forward pass and (d) implementing constant multiplication with multi-valued memory devices in the backward passes. With such modification, identifying make and break monomials with inverted variable inputs requires comparing dot products of the forward pass with fixed values of (b) 0 and (c) 1, correspondingly. (e) Increasing throughput by implementing backward passes with separate crossbar memory arrays. On panel a, values in blue correspond to the inverted version of the same variable assignment x_1=1, x_2=0, x_3=1, x_4=0 as that was used in Fig. 1a of main text. The polynomial mapped to the crossbars in all panels is the same as that in Fig. 1a of main text.

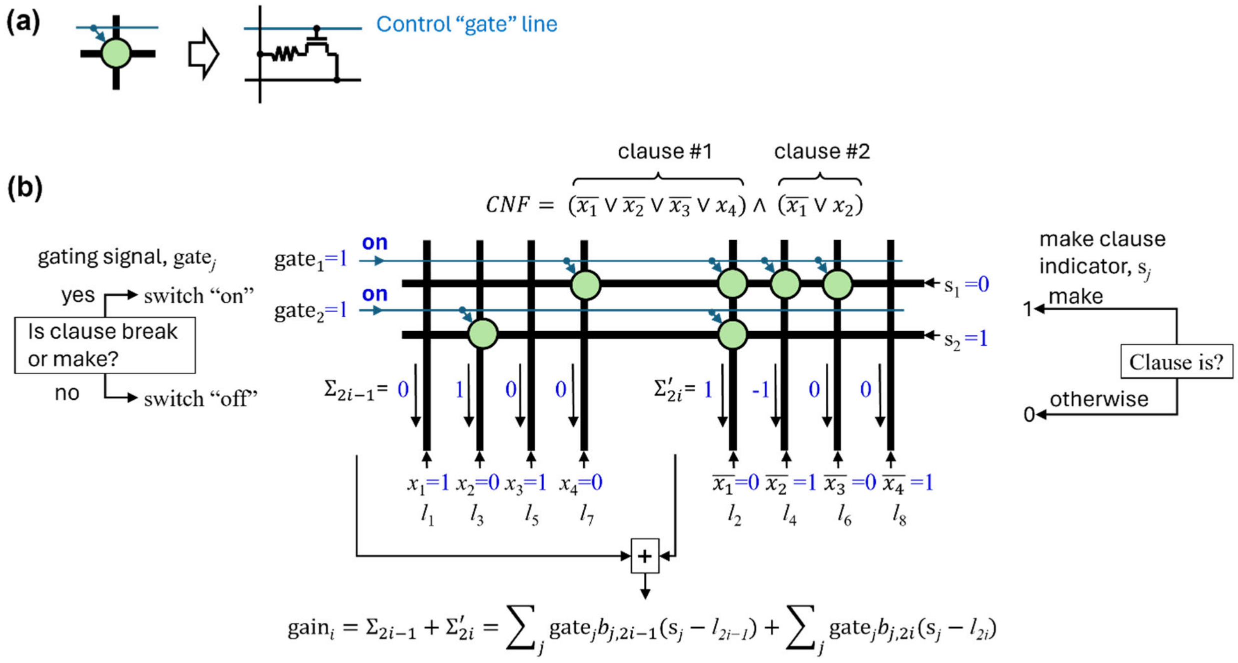

Figure S5. Extension for three-terminal memory devices: (a) A three-terminal memory device based on 1T1R memory. (b) Backward pass implementation to compute gain values using a single M×2N crossbar array (instead of two such arrays as shown in Figs. 2d and 2e), where M is the number of clauses and N is the number of variables. Values in blue correspond to the specific variable assignment x_1=1, x_2=0, x_3=1, x_4=0. Σ_{2i-1} and Σ'_{2i} are sums of triple products along (2i-1)-th and 2i-th columns corresponding to variable x_i's normal and complementary literals. Specifically, the triple product in Σ_{2i-1} (Σ'_{2i}) consists of terms $s_j - l_{2i-1}$ ($s_j - l_{2i}$), where s_j is the make clause indicator, the weight value $b_{j,2i-1}$ ($b_{j,2i}$), and gating signal gate$_j$. The gating signals are applied to 1T1R control lines and turn on only make or break type clauses. Variable gain values are obtained by adding Σ_{2i-1} and Σ'_{2i} - see equation (47) of Supplementary Note 4 for more details.

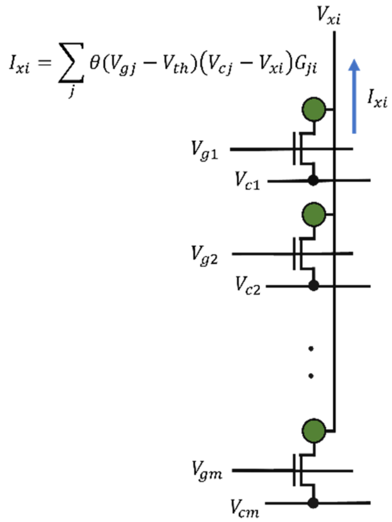

Figure S6. Single bit line in a 1T1R memristor crossbar array. The transistor in each cell acts like a switch, mathematically represented by the shifted Heaviside step function $\theta(V_{gj}-V_{th})$. When the switch is closed ($V_{gj}>=V_{th}$), a current proportional to the product of the voltage difference across the cell and the conductance of the memristor flows. When the switch is open ($V_{gj}<V_{th}$), zero current flows.

```
p cnf 14  64
 5 10 13 0
3 -11 -12 0
7 -8 -12 0
0 -2 -7 0
-6 -7 13 0
2 -8 -11 0
5 7 -9 0
-6 -7 -11 0
-5 -7 8 0
0 -6 -12 0
2 4 9 0
1 -7 9 0
7 -8 -10 0
-1 5 12 0
-3 -6 13 0
0 1 -12 0
-1 6 10 0
1 5 -10 0
-1 -11 -13 0
2 4 -8 0
5 -6 -10 0
1 -6 13 0
9 10 13 0
0 5 10 0
2 5 11 0
-1 -2 5 0
0 -8 9 0
-8 -11 -12 0
10 -11 12 0
0 -2 13 0
0 -4 -6 0
-4 -5 -12 0
1 2 -11 0
2 -3 -13 0
11 12 -13 0
0 7 13 0
2 6 -8 0
-2 -6 7 0
-2 7 -12 0
0 -4 -10 0
-4 -6 -11 0
1 -2 -13 0
3 8 -11 0
-11 12 -13 0
-1 -4 8 0
2 -4 -11 0
-3 -8 10 0
-1 6 13 0
1 6 11 0
4 -10 11 0
0 -10 12 0
0 2 -11 0
9 11 -13 0
-1 -4 10 0
0 4 -10 0
4 9 -10 0
0 3 8 0
0 -1 -13 0
0 9 -12 0
1 11 -12 0
-4 8 11 0
2 -5 6 0
-1 5 -7 0
3 5 13 0
%
0
```

Figure S7. 3-SAT instance solved in the experimental demo.

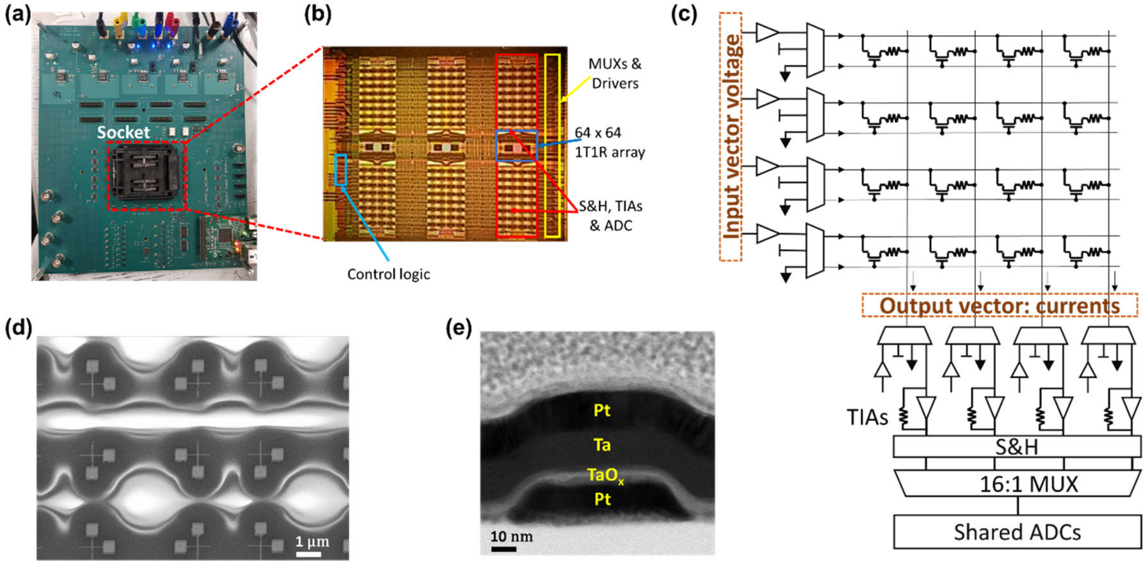

Figure S8. Experimental setup. Hewlett Packard Enterprise’s CMOS/memristor prototyping setup[22]. (a) Photo of the printed circuit board used for testing the chip. (b) Photo of a CMOS die hosting three 1T1R memristor crossbar arrays and peripheral circuitry. (c) Circuit schematic of a single crossbar and its peripherals from the CMOS/memristor chip. (d) Scanning Electron Microscope (SEM) image of the memristor devices and (e) cross-section Transmission Electron Microscopy (TEM) image of the Pt/Ta/TaO_x/Pt memristor stack.

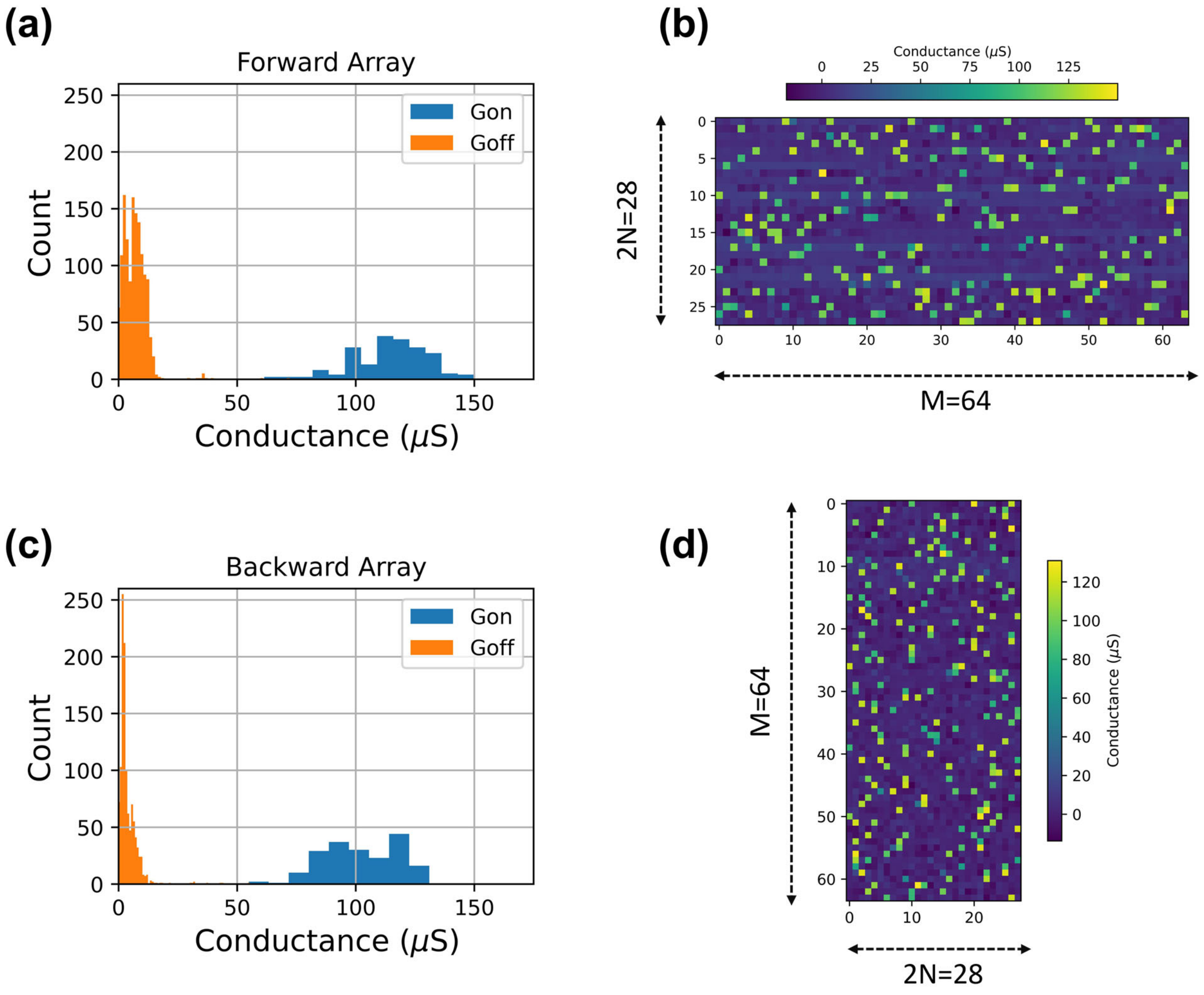

Figure S9. Memristor tuning statistics. Histogram of tuned memristor conductance in forward and backward arrays (a), (c). Conductance map of the tuned forward and backward crossbar arrays (b), (d). The mean and standard deviation of ON state memristors are 108.3μS and 16μS respectively, whereas that of OFF state memristors are 3.3μS and 6μS respectively.

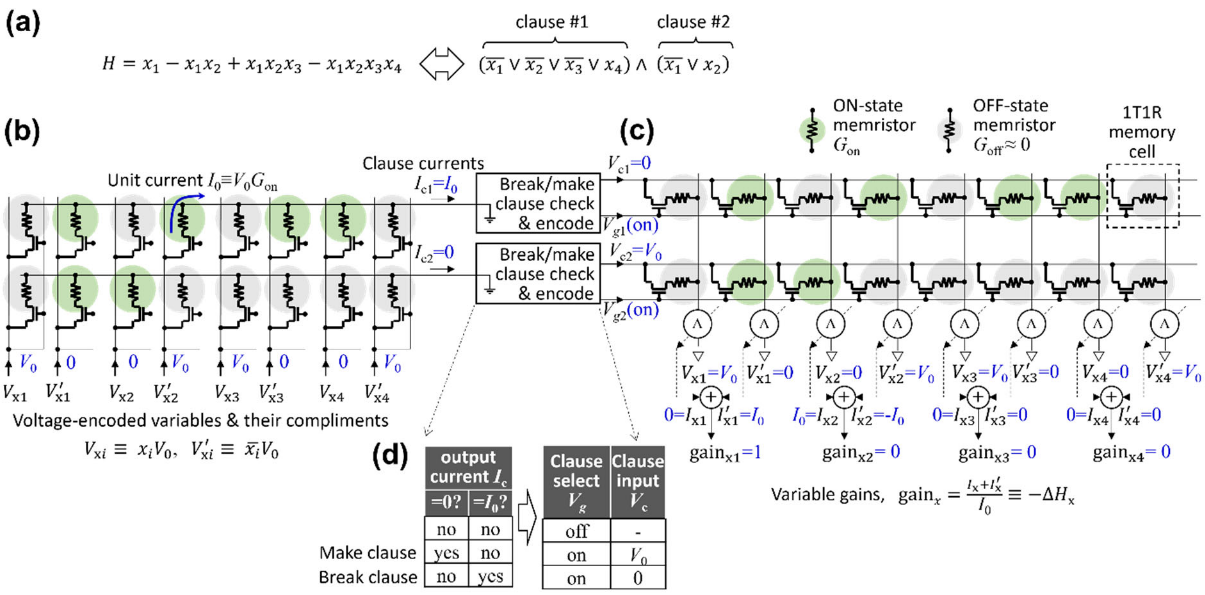

Figure S10. A compact 1T1R implementation for CNF-form Boolean function gain calculation. (a) The considered toy example of CNF function. (b, c) 1T1R circuit implementations of (b) forward and (c) backward computation of the proposed approach. (d) Peripheral logic functionality. The bit lines in the second array are tied to the literal values so that $I_x + I'_x$ is proportional to the $\text{gain}_{xi} = \sum_j \text{gate}_j b_{j,2i-1}(s_j - l_{2i-1}) + \sum_j \text{gate}_j b_{j,2i}(s_j - l_{2i})$ – see Fig. S5, its discussion, and Supplementary Note 4-5. Panels a and b are the same as in Fig. 2a and are shown for convenience. Also, note that backward pass in such an approach requires applying different voltages to the word, control and bit lines of the array, which cannot be implemented in the utilized experimental setup.

	Benchmark problem	# var (N)	# clauses (M')	Clause /Var (M'/N)	Maximum order Kmax	Averare order <K>	# var after converting to QUBO	# weights in proposed approach	# weights in QUBO with Rosenberg mapping	Ratio
SATLIB	uf20-01.cnf	20	91	4.55	3	3.00	111	7.28E+03	2.46E+04	3.38
	uf50-01.cnf	50	218	4.36	3	3.00	268	4.36E+04	1.44E+05	3.29
	uf75-01.cnf	75	325	4.33	3	3.00	400	9.75E+04	3.20E+05	3.28
	uf100-01.cnf	100	430	4.30	3	3.00	530	1.72E+05	5.62E+05	3.27
	uf125-01.cnf	125	538	4.30	3	3.00	663	2.69E+05	8.79E+05	3.27
	uf150-01.cnf	150	645	4.30	3	3.00	795	3.87E+05	1.26E+06	3.27
	uf175-01.cnf	175	753	4.30	3	3.00	928	5.27E+05	1.72E+06	3.27
	uf200-01.cnf	200	860	4.30	3	3.00	1060	6.88E+05	2.25E+06	3.27
	uf225-01.cnf	225	960	4.27	3	3.00	1185	8.64E+05	2.81E+06	3.25
	uf250-01.cnf	250	1065	4.26	3	3.00	1315	1.07E+06	3.46E+06	3.25
XOR	xor3.cnf	3	4	1.33	3	3.00	7	4.80E+01	9.80E+01	2.04
	xor4.cnf	4	8	2.00	4	4.00	28	1.28E+02	1.57E+03	12.25
	xor5.cnf	5	16	3.20	5	5.00	85	3.20E+02	1.45E+04	45.16
	xor6.cnf	6	32	5.33	6	6.00	230	7.68E+02	1.06E+05	137.76
	xor7.cnf	7	64	9.14	7	7.00	583	1.79E+03	6.80E+05	379.34
	xor8.cnf	8	128	16.00	8	8.00	1416	4.10E+03	4.01E+06	979.03
	xor9.cnf	9	256	28.44	9	9.00	3337	9.22E+03	2.23E+07	2416.57
	xor10.cnf	10	512	51.20	10	10.00	7690	2.05E+04	1.18E+08	5775.01
SAT2020	battleship-3-5-sat.cnf	15	24	1.60	5	3.13	60	1.44E+03	7.20E+03	5.00
	battleship-4-7-sat.cnf	28	58	2.07	7	3.38	172	6.50E+03	5.92E+04	9.11
	battleship-5-9-sat.cnf	45	115	2.56	9	3.52	370	2.07E+04	2.74E+05	13.23
	battleship-6-10-sat.cnf	60	186	3.10	10	3.55	600	4.46E+04	7.20E+05	16.13
	battleship-6-11-sat.cnf	66	201	3.05	11	3.61	678	5.31E+04	9.19E+05	17.33
	Steiner-9-4-bce.cnf	63	55	0.87	3	2.56	95	1.39E+04	1.81E+04	1.30
	Steiner-15-6-bce.cnf	165	146	0.88	3	2.60	254	9.64E+04	1.29E+05	1.34
	sgen1-sat-100-100.cnf	100	240	2.40	5	2.50	300	9.60E+04	1.80E+05	1.88
	sgen3-n120-s12930489-sat.cnf	120	288	2.40	5	2.50	360	1.38E+05	2.59E+05	1.88
	sgen1-sat-120-100.cnf	120	288	2.40	5	2.50	360	1.38E+05	2.59E+05	1.88
	sgen1-sat-140-100.cnf	140	336	2.40	5	2.50	420	1.88E+05	3.53E+05	1.88
	sgen4-sat-160-8.cnf	160	384	2.40	5	2.50	480	2.46E+05	4.61E+05	1.88
	sgen1-sat-160-100.cnf	160	384	2.40	5	2.50	480	2.46E+05	4.61E+05	1.88
	driverlog1_ks99i.renamed-as.sat05-3951.cnf	207	588	2.84	4	2.08	281	4.87E+05	1.58E+05	0.32
	mod2-rand3bip-sat-210-2.sat05-2159.reshuffled-07.cnf	210	840	4.00	3	3.00	1050	7.06E+05	2.21E+06	3.13
	mod2-rand3bip-sat-220-2.sat05-2174.reshuffled-07.cnf	220	880	4.00	3	3.00	1100	7.74E+05	2.42E+06	3.13
	mod2-rand3bip-sat-240-3.sat05-2205.reshuffled-07.cnf	240	960	4.00	3	3.00	1200	9.22E+05	2.88E+06	3.13
	driverlog3_v01a.renamed-as.sat05-3963.cnf	170	1559	9.17	5	2.14	472	1.06E+06	4.46E+05	0.42
	mod2c-rand3bip-sat-190-3.sat05-2445.reshuffled-07.cnf	271	1972	7.28	6	4.48	8091	2.14E+06	1.31E+08	61.25
	20180322_164245263_p_cnf_320_1120.cnf	320	1120	3.50	3	3.00	1440	1.43E+06	4.15E+06	2.89
	289-sat-11x4.cnf	176	1628	9.25	4	3.68	4268	1.15E+06	3.64E+07	31.79
	289-sat-4x8.cnf	128	896	7.00	4	3.57	2240	4.59E+05	1.00E+07	21.88
	289-sat-5x8.cnf	160	1400	8.75	4	3.66	3640	8.96E+05	2.65E+07	29.58
	289-sat-6x8.cnf	192	2016	10.50	4	3.71	5376	1.55E+06	5.78E+07	37.33
	289-sat-6x9.cnf	216	2538	11.75	4	3.74	6858	2.19E+06	9.41E+07	42.90
	289-sat-7x6.cnf	168	1554	9.25	4	3.68	4074	1.04E+06	3.32E+07	31.79
	**7cnf20_90000_90000_7.shuffled.cnf**	**20**	**1532**	**76.60**	**7**	**7.00**	**13808**	**1.23E+05**	**3.81E+08**	**3111.31**
	aes_32_1_keyfind_1.cnf	300	1016	3.39	5	3.05	1656	1.22E+06	5.48E+06	4.50
	aes_32_1_keyfind_2.cnf	300	1016	3.39	5	3.05	1656	1.22E+06	5.48E+06	4.50
JHN	jnh1.cnf	100	850	8.50	14	5.17	4697	3.40E+05	4.41E+07	129.78
	jnh2.cnf	100	850	8.50	10	4.93	4311	3.40E+05	3.72E+07	109.32
	jnh3.cnf	100	850	8.50	11	4.90	4265	3.40E+05	3.64E+07	107.00
SEMIPRIME	semiprime15.cnf	22	73	3.32	3	2.86	90	6.42E+03	1.62E+04	2.52
	semiprime21.cnf	52	187	3.60	3	2.93	232	3.89E+04	1.08E+05	2.77
	semiprime55.cnf	68	248	3.65	3	2.94	308	6.75E+04	1.90E+05	2.81
	semiprime91.cnf	116	434	3.74	3	2.95	540	2.01E+05	5.83E+05	2.90
	semiprime221.cnf	138	519	3.76	3	2.96	646	2.86E+05	8.35E+05	2.91
	semiprime323.cnf	204	777	3.81	3	2.97	968	6.34E+05	1.87E+06	2.96
	semiprime651.cnf	232	886	3.82	3	2.97	1104	8.22E+05	2.44E+06	2.96
	semiprime1271.cnf	316	1216	3.85	3	2.97	1516	1.54E+06	4.60E+06	2.99
	semiprime8633.cnf	492	1908	3.88	3	2.98	2380	3.75E+06	1.13E+07	3.02
	semiprime60491.cnf	658	2563	3.90	3	2.98	3198	6.75E+06	2.05E+07	3.03

Figure S11. Area advantage for the studied benchmarks. The highlighted row corresponds to the largest area advantage for the SAT2020 industrial benchmark problem.

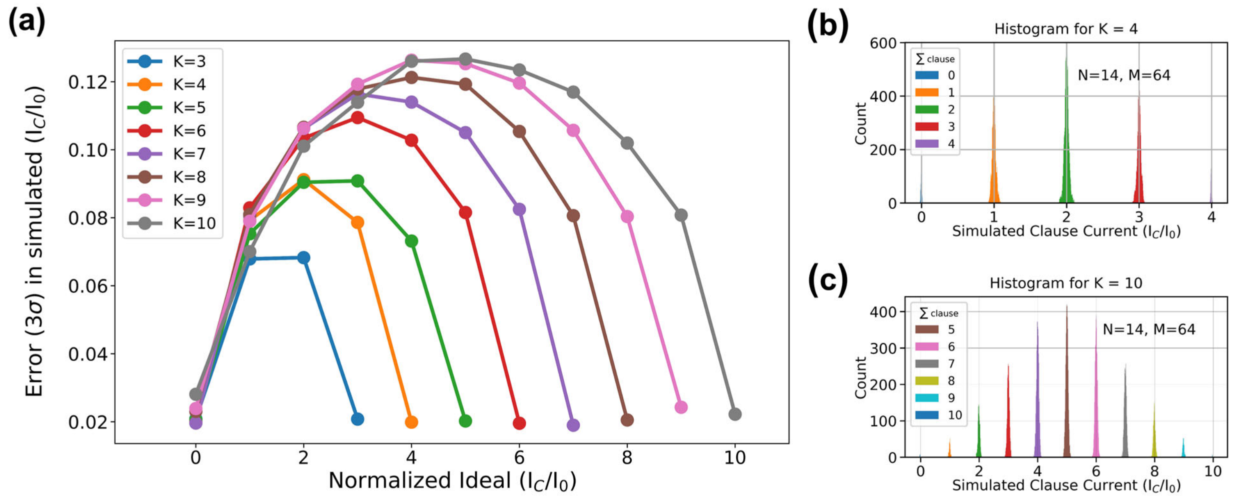

Figure S12. Error in clause current in forward array for different values of *K* based on SPICE simulation. (a) Plot of three times standard deviation in the simulated normalized clause current for different values of ideal normalized clause current at different values of K (maximum order of SAT CNF mapped to the crossbar). (b-c) Histogram of the simulated clause current values for (b) $K = 4$ and (c) $K = 10$. Each K-SAT problem mapped to the 64×64 forward array crossbar has $N = 14$, $M = 64$ and was applied 500 randomly generated literal vector assignments. Memristor conductance were drawn from normal distribution with mean set to G_{on} = 100μS and G_{off} = 1μS and standard deviations $\sigma_{Gon} = 3\mu S$, $\sigma_{Goff} = 0.25\mu S$. See Supplementary Note 6 for details on simulation setup.

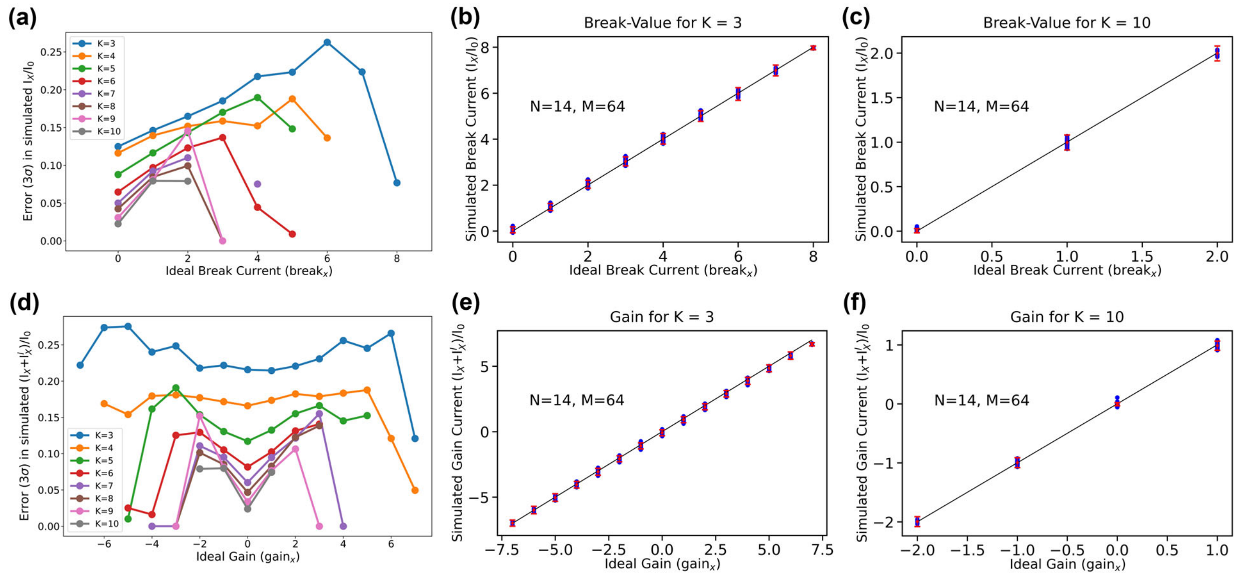

Figure S13. Error in break and gain currents in backward array for different values of *K* based on SPICE simulation. (a, d) Plot of three times standard deviation in the simulated normalized (a) break and (d) gain currents for different values of ideal normalized break and gain currents respectively at different values of *K* (maximum order of the SAT CNF mapped to the crossbar). (b, c, e, f) Simulated vs ideal break current values for (b) $K = 3$ and (c) $K = 10$ and simulated vs ideal gain current values for (e) $K = 3$ and (f) $K = 10$. Each K-SAT problem mapped to the 64×64 backward array crossbar has $N = 14$, $M = 64$. The crossbar received clause select (V_g) and input (V_c) signals (Fig. S10) corresponding to 500 randomly generated literal vector assignments. Memristor conductance were drawn from normal distribution with mean set to G_{on} = 100µS and G_{off} = 1µS and standard deviations $\sigma_{Gon} = 3\mu S$, $\sigma_{Goff} = 0.25\mu S$. See Supplementary Note 6 for details on simulation setup.

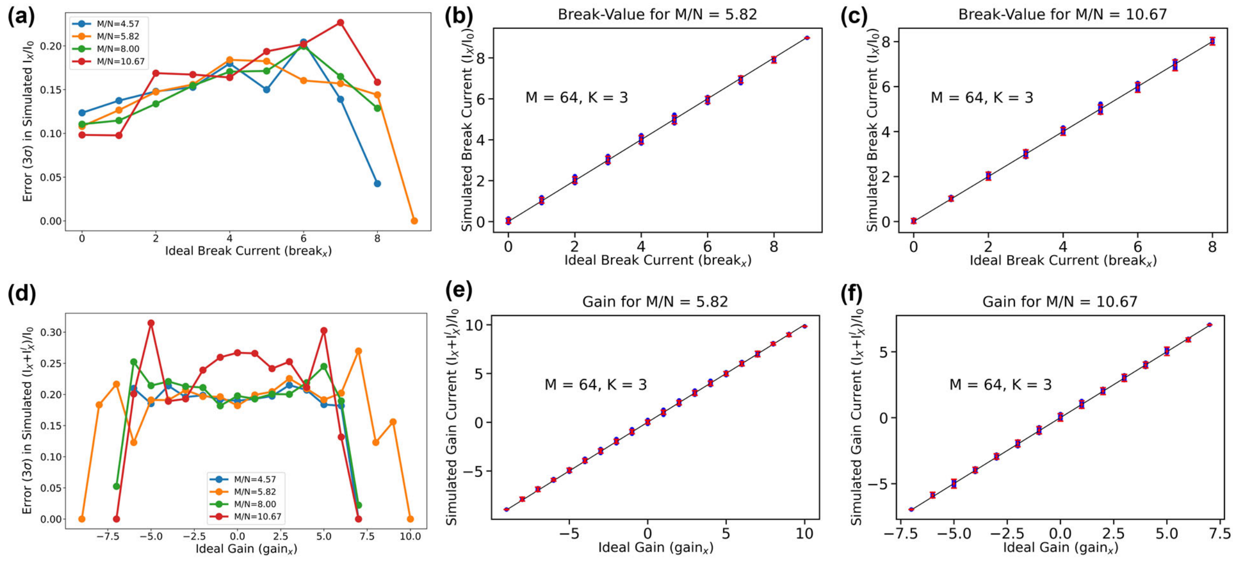

Figure S14. Error in break and gain currents in backward array for different values of clause-to-variable ratio based on SPICE simulation. (a, d) Plot of three times standard deviation in the simulated normalized (a) break and (d) gain currents for different values of ideal normalized break and gain currents respectively at different values of clause-to-variable ratio (M/N) of the SAT CNF mapped to the crossbar. (b, c, e, f) Simulated vs ideal break current values for (b) $M/N = 5.82$ and (c) $M/N = 10.67$ and simulated vs ideal gain current values for (e) $M/N = 5.82$ and (f) $M/N = 10.67$. Each K-SAT problem mapped to the 64×64 backward array crossbar has $K = 3$ and $M = 64$. The crossbar received clause select (V_g) and input (V_c) signals (Fig. S10) corresponding to 500 randomly generated literal vector assignments. Memristor conductance were drawn from normal distribution with mean set to G_{on} = 100μS and G_{off} = 1μS and standard deviations σ_{Gon} = 3μS, σ_{Goff} = 0.25μS. See Supplementary Note 6 for details on simulation setup.

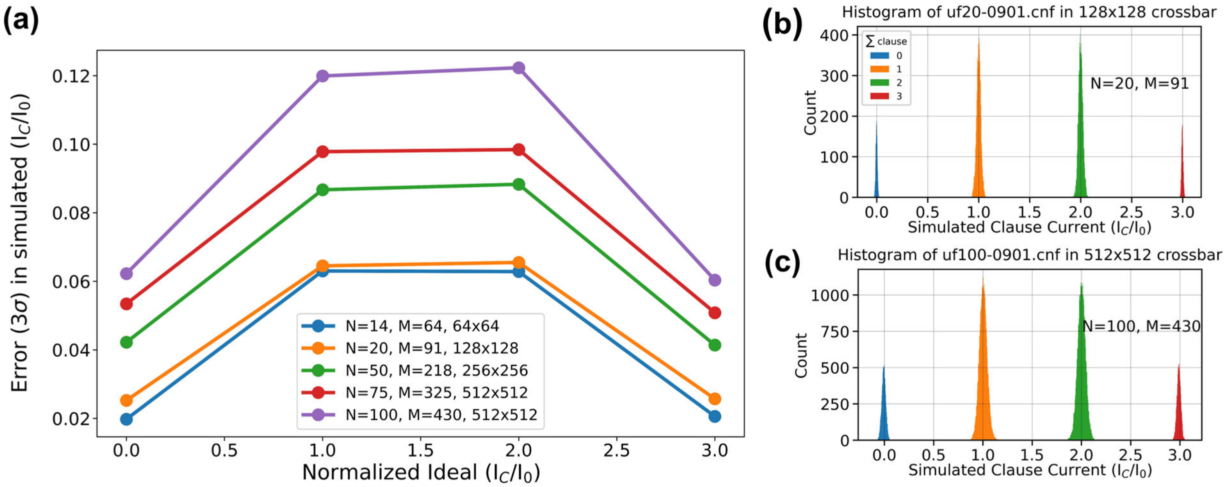

Figure S15. Error in clause current in forward array for different crossbar dimensions based on SPICE simulation. (a) Plot of three times standard deviation in the simulated normalized clause current for different values of ideal normalized clause current at different forward crossbar array sizes. (b-c) Histogram of the simulated clause current values for 3-SAT problems with (b) $N = 20$, $M = 91$ and (c) $N = 100$, $M = 430$ mapped to crossbar arrays with dimensions 128×128 and 512×512, respectively. For each 3-SAT problem, 200 randomly generated literal vector assignments were applied as inputs. Random 3-SAT instance used for experiments was mapped to the 64×64 crossbar, whereas instances uf20-0901, uf50-0901, uf75-051 and uf100-0901 from SATLIB benchmark[46] were mapped to crossbars with linear dimensions ranging from 128 to 512. Memristor conductance were drawn from normal distribution with mean set to G_{on} = 100μS and G_{off} = 1μS and standard deviations $\sigma_{\mathrm{Gon}} = 3\mu S$, $\sigma_{\mathrm{Goff}} = 0.25\mu S$. See Supplementary Note 6 for details on simulation setup.

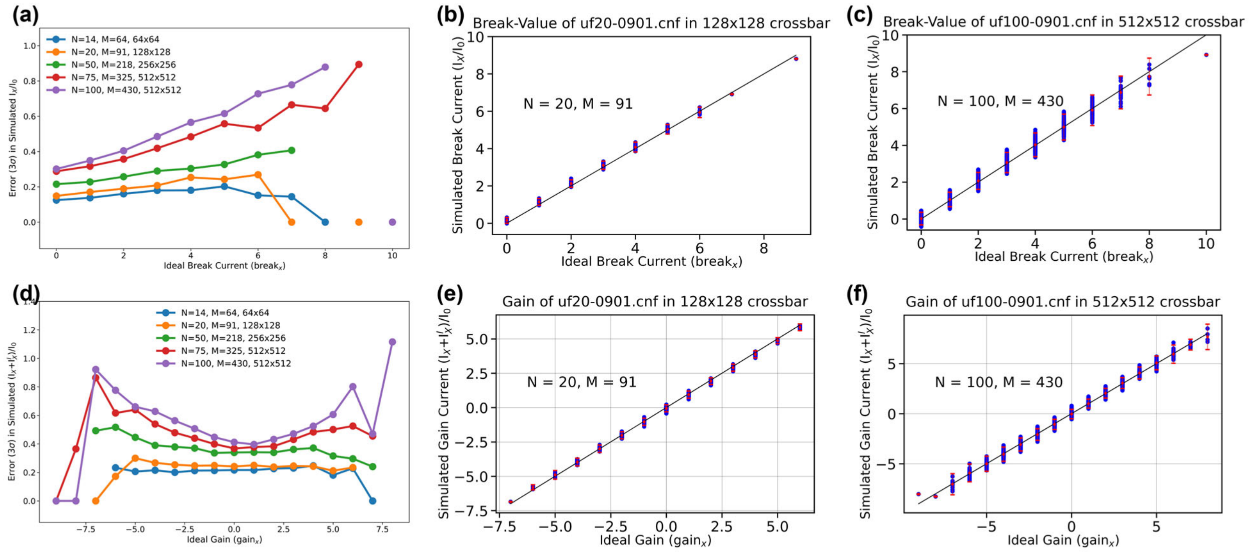

Figure S16. Error in break and gain currents in backward array for different crossbar dimensions based on SPICE simulation. (a, d) Plot of three times standard deviation in the simulated normalized (a) break and (d) gain currents for different values of ideal normalized break and gain currents respectively at different forward crossbar array sizes. (b, c, e, f) Simulated vs ideal (b, c) break and (e ,f) gain current values for 3-SAT problems with (b, e) N = 20, M = 91 and (c, f) N = 100, M = 430 mapped to crossbar arrays with dimensions 128×128 and 512×512, respectively. Random 3-SAT instance used for experiments was mapped to the 64×64 crossbar, whereas instances uf20-0901, uf50-0901, uf75-051 and uf100-0901 from SATLIB benchmark[46] were mapped to crossbars with linear dimensions ranging from 128 to 512. Crossbar arrays received clause select (V_g) and input (V_c) signals (Fig. S10) corresponding to 200 randomly generated literal vector assignments. Memristor conductance were drawn from normal distribution with mean set to G_{on} = 100μS and G_{off} = 1μS and standard deviations σ_{Gon} = 3μS, σ_{Goff} = 0.25μS. See Supplementary Note 6 for details on simulation setup.

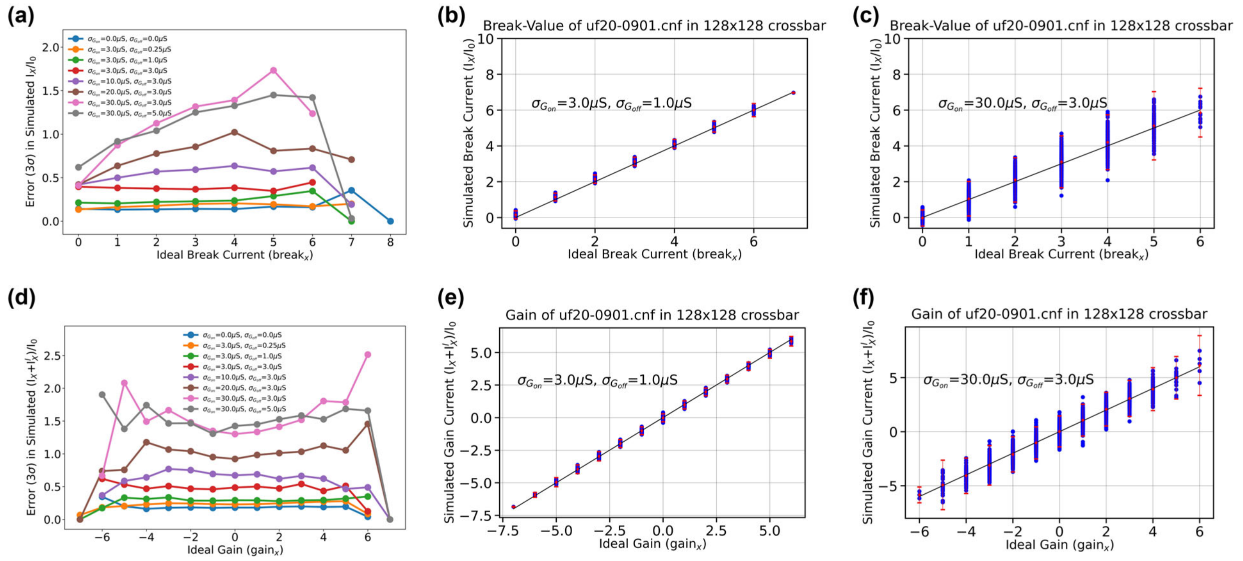

Figure S17. Error in break and gain currents in backward array for different levels of memristor programming error, based on SPICE simulation. (a, d) Plot of three times standard deviation in the simulated normalized (a) break and (d) gain currents for different values of ideal normalized break and gain currents respectively at different levels of memristor programming error. (b, c, e, f) Simulated vs ideal (b, c) break and (e, f) gain current values for 3-SAT problem mapped to 128×128 crossbar array with (b, e) $\sigma_{\mathrm{Gon}} = 3\mu\mathrm{S}$, $\sigma_{\mathrm{Goff}} = 1\mu\mathrm{S}$ and (c, f) $\sigma_{\mathrm{Gon}} = 30\mu\mathrm{S}$, $\sigma_{\mathrm{Goff}} = 3\mu\mathrm{S}$. Uniform random 3-SAT instance uf20-0901 from SATLIB benchmark[46] was used. Crossbar arrays received clause select (V_{g}) and input (V_{c}) signals (Fig. S10) corresponding to 200 randomly generated literal vector assignments. See Supplementary Note 6 for details on simulation setup.

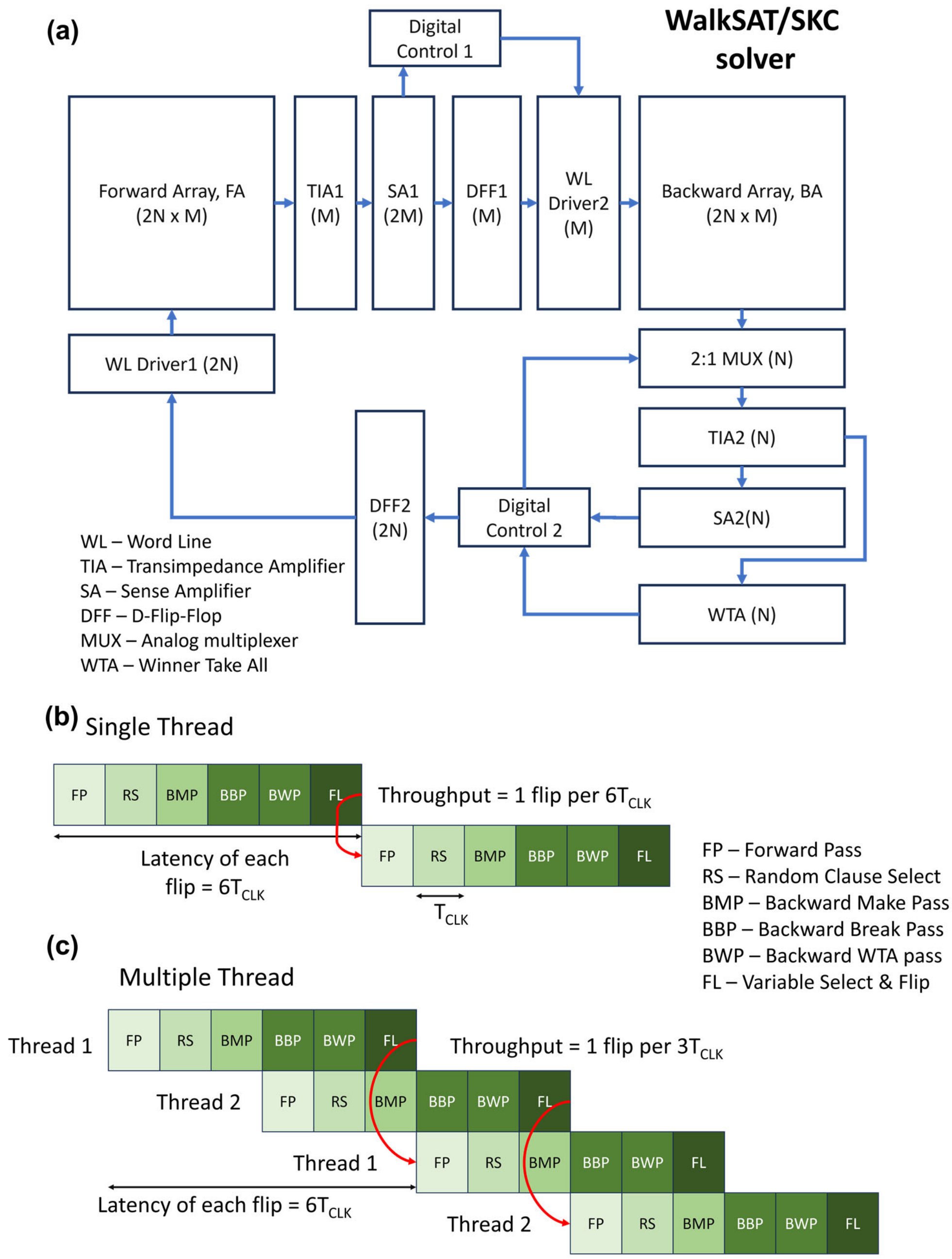

Figure S18. Hardware description of WalkSAT/SKC solver. (a) Block level diagram of circuit components required for implementation of a WalkSAT/SKC solver. The number inside brackets denote the count/dimension of the circuit block. See Table S1 for description and energy, area, latency details of each block. (b, c) Timing diagrams of the solver when (b) single and (c) two threads are run. Six pipeline stages are required for a single variable flip in any thread. See Table S2 for description and the active hardware components of each stage.

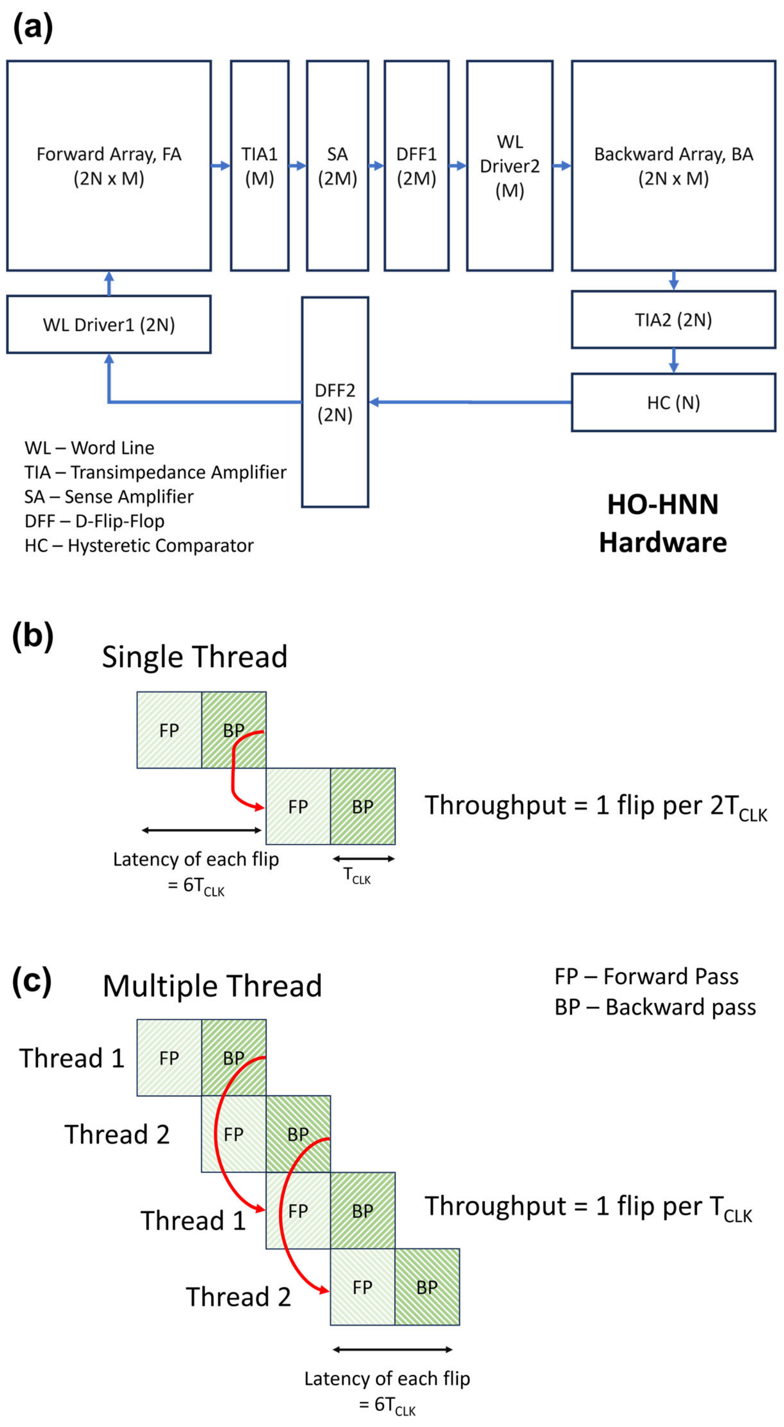

Figure S19. Hardware description of HO-HNN solver. (a) Block level diagram of circuit components required for implementation of a High Order HNN solver. The number inside brackets denote the count/dimension of the circuit block. See Table S3 for description and energy, area, latency details of each block. (b, c) Timing diagrams of the solver when (b) single and (c) two threads are run. Two pipeline stages are required for a single variable flip in any thread. See Table S4 for description and the active hardware components of each stage.

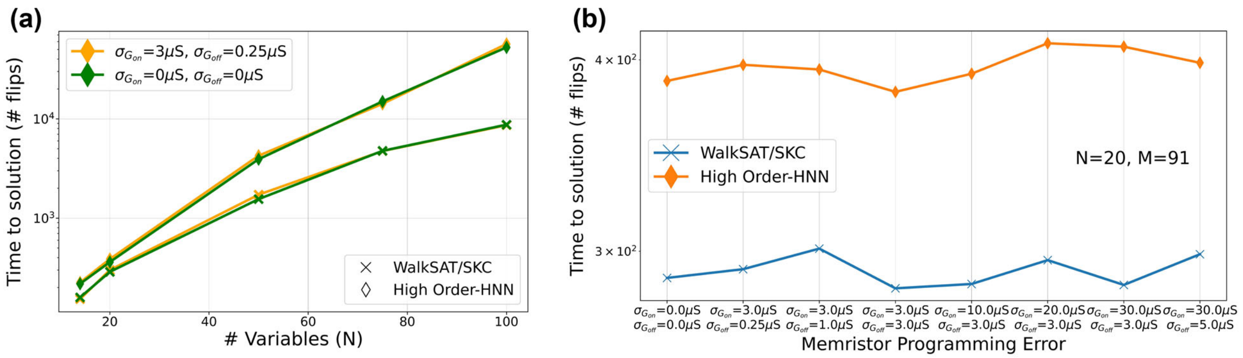

Figure S20. Hardware-aware simulations on uniform random 3-SAT problems. (a) Scaling trend of time to solution (in number of variable flips) for WalkSAT/SKC and High Order HNN algorithms with problem size, for ideal and non-ideal memristor programming. (b) Plot of time to solution for different levels of memristor programming error on 20-variable 91-clause uniform random 3-SAT problem instances.

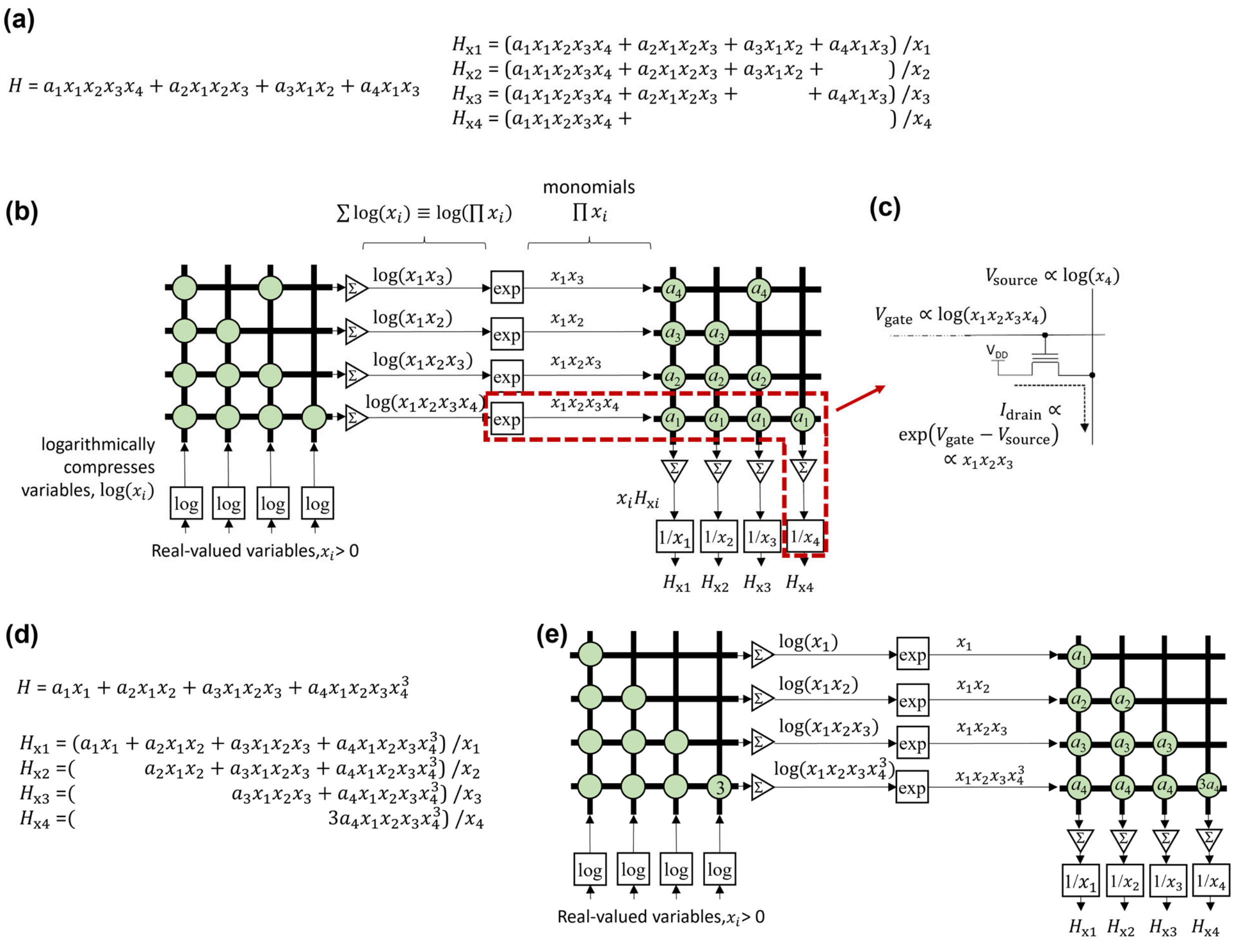

Figure S21. Gradient computation of real-valued functions: (a) Considered multi-linear polynomial energy function of real-valued variables and its partial derivatives. (b) The main idea and (c) circuit implementation with floating gate memories. In such an implementation, the exponentiation and division by variable ($1/x$) blocks are replaced with coupling weights consisting of floating gate memories operating in a subthreshold regime. Logarithmically encoded monomial values from the first array and log-encoded variable values are directly applied to the second array's gate and source voltage lines. As a result, division and exponentiation operations are executed by virtue of the exponential dependence of cell current on the difference of applied gate and source voltages. (d) Energy function and its partial derivatives and (e) crossbar circuit implementation for a more general polynomial (non-multi-linear). Note that a term with x_4 in the third power of the energy function is implemented with multi-state weights in the first crossbar and factor of 3 in the second crossbar.

(a)

$$H = a_1 x_1 x_2 x_3 x_4 + a_2 x_1 x_2 x_3 + a_3 x_1 x_2 + a_4 x_1 x_3$$

(b)

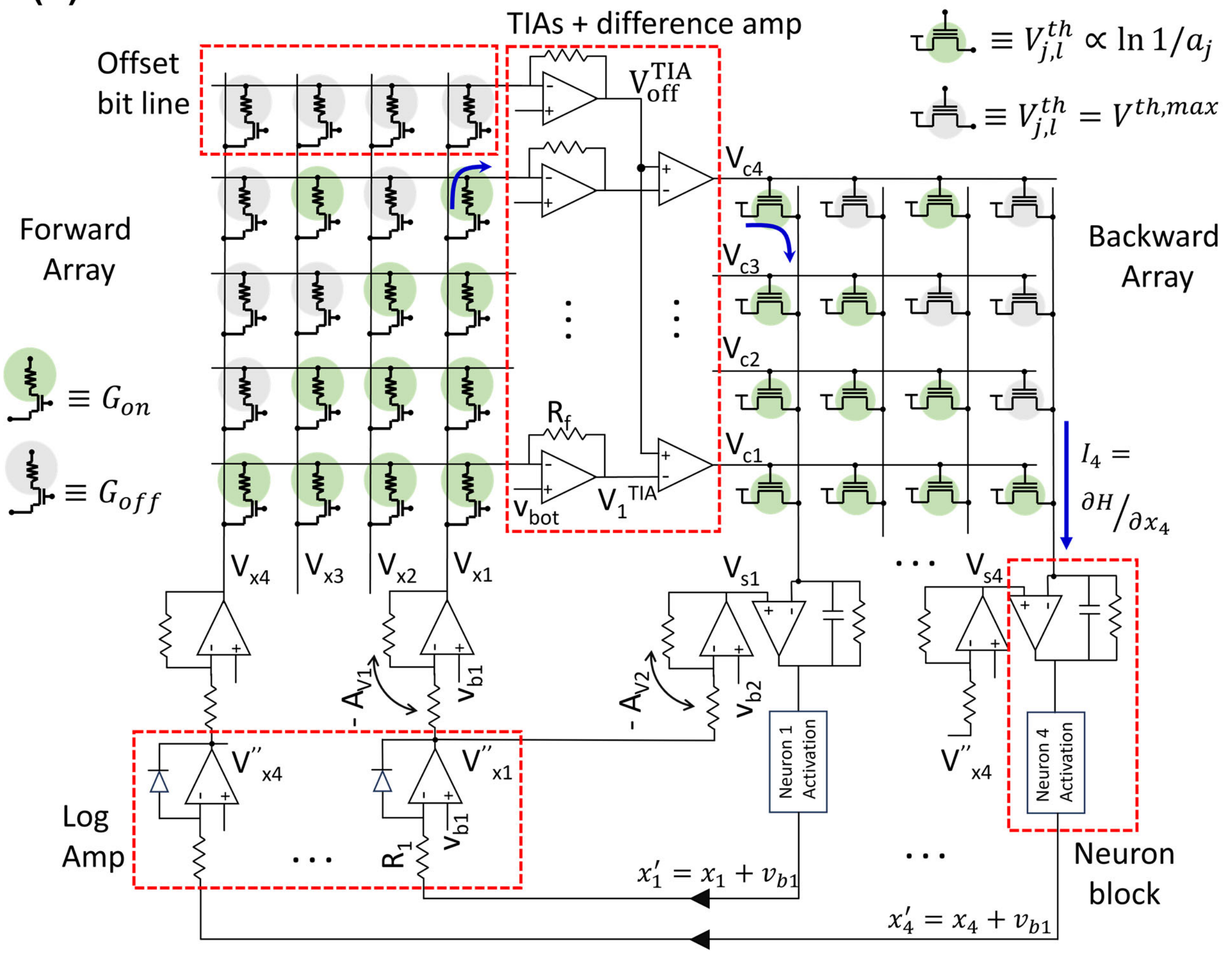

Figure S22. Possible circuit implementation of real-valued gradient computing hardware. (a) Considered multi-linear polynomial energy function of real-valued variables and (b) possible circuit implementation of its real-valued gradient computing hardware. Forward and backward arrays comprise of linear and exponential crosspoint devices: memristors operating in its linear regime and floating-gate memory cells operating in its subthreshold regime respectively.

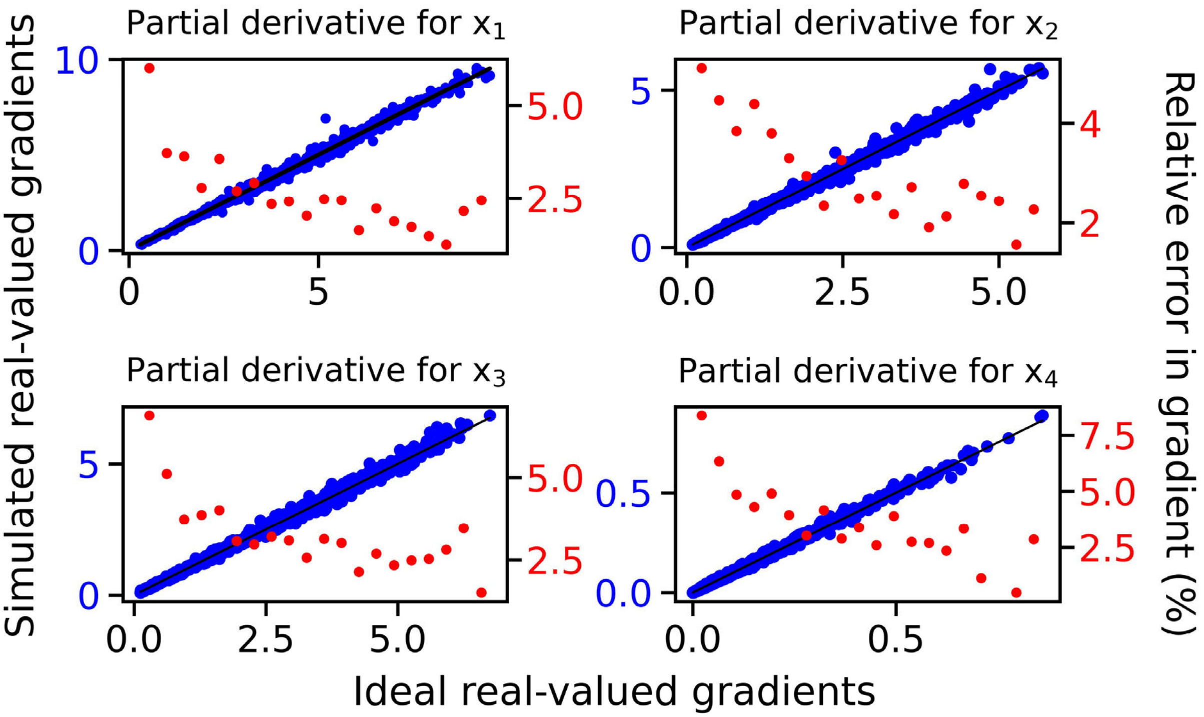

Figure S23. Simulation result of real-valued gradient computation hardware. The left axis shows plot of the simulated real-valued gradient depicted by the bit line current in the backward array vs ideal gradient values. The right axis shows plot of the relative error in the gradient values. The error is larger for smaller gradients mainly due to the assumed fixed absolute tuning error for floating gate memories, i.e., larger relative error for smaller weights in this modeling study.

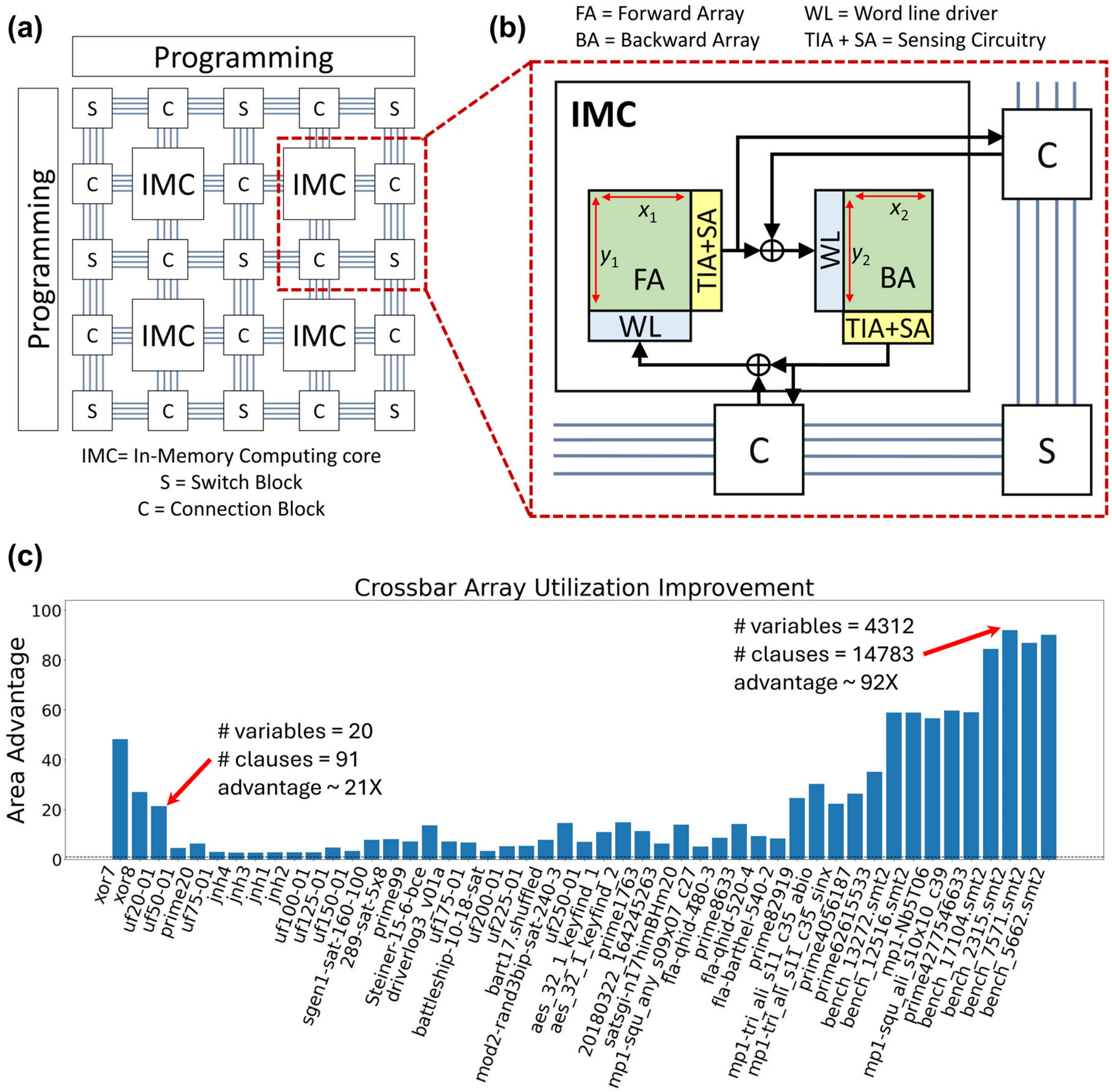

Figure S24. Multi-tile architecture for high-degree gradient computation. (a) Architecture inspired by Field Programmable Gate Arrays (FPGAs) for implementing large-scale high order Ising machines, comprising of In-Memory Computing (IMC) cores and routing fabric that includes switch and connection blocks. (b) Detailed description of a single tile, where the IMC core contains two crossbar arrays: forward and backward and their associated peripheral circuitry. (c) Compression achieved in total crossbar array area when large-scale high-degree optimization problems are divided into multiple tiles using FPGA-inspired packing.

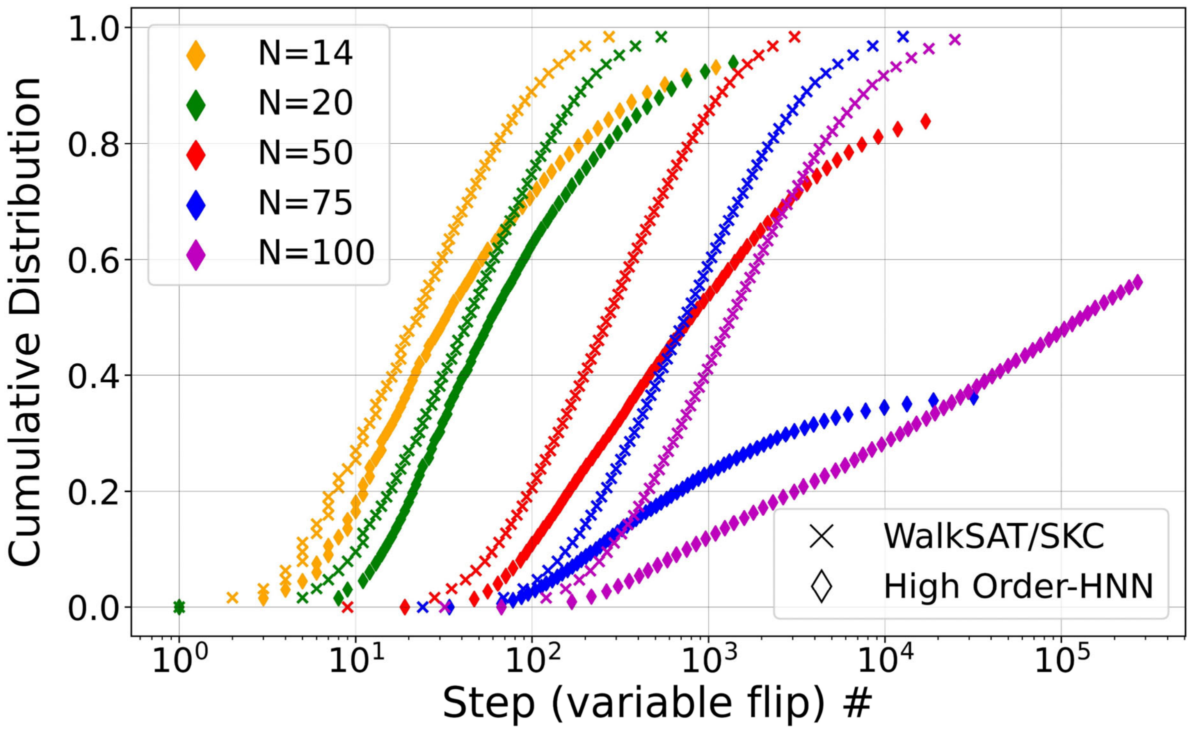

Figure S25. Hardware-aware algorithmic simulations. Cumulative distribution function for the number of variable flips required by WalkSAT/SKC and high-order Hopfield Neural Network algorithms to reach global minimum across all iterations (up to MAX_ITER) for all instances of 3-SAT problems with varying size. The number of instances for problems with N = 14, 20, 50, 75 and 100 were 80, 80, 80, 50 and 80 respectively. For both algorithms, MAX_ITER was set to 500. The total number of datapoints for each problem size and a specific algorithm used to generate the plot is equal to the number of instances of that problem size times MAX_ITER, which is 25,000 for N = 75 and 40,000 for all others. However, only 63 data points sampled regularly from the sorted list of run-length distribution of each problem size across all its instances and iterations per instance, are plotted for clarity. Note that datapoints correspond to successful iterations only. Hardware-aware algorithmic simulations are taking into accounts errors in break/gain values due to memristor tuning errors, with the assumed three times standard deviation for the conductance tuning of $\sigma_{\text{Gon}} = 3\mu\text{S}$, $\sigma_{\text{Goff}} = 0.25\mu\text{S}$.

Component (Description)	Phase (Shorthand)	Count / Dimension	Latency (ns)	Energy (pJ per unit of component per cycle)	Area (μm^2 per unit of component)
Forward/Backward word line driver	WL1 / WL2	2N / M	0.501	0.27	2.73
Forward/Backward Array	FA/BA	2N x M	<0.5	0.008(ON), 0.00008(OFF)	0.0123
Forward/Backward transimpedance amplifier	TIA1/TIA2	M / N	0.3	0.26	10.5135
Forward/Backward sense amplifier	SA1/SA2	2M / N	<0.09	0.0023	8.058
Forward/Backward register array	DFF1/DFF2	M / 2N	<0.1	0.023	1.915
2-to-1 Multiplexer	MUX	N	0.0073	0.00023	2.084
Winner-take-all circuit	WTA	N	0 - 2	0.037	29.01

Table S1. Area and energy breakdown for circuit components of WalkSAT/SKC hardware based on 32nm technology node.

Phase (Shorthand)	Phase (Name)	Active Components
FP	Forward Pass	WL1(2N), FA, TIA1(M), SA1(2M)
RS	Random Clause Select	DFF1(M)
BMP	Backward Make Pass	WL2(1), BA, MUX(N), TIA2(N), SA2(N)
BBP	Backward Break Pass	WL2(M), BA, MUX(N), TIA2(k), SA2(k)
BWP	Backward WTA Pass	BA, TIA2(k), WTA(N)
FL	Variable Select & Flip	DFF2(2N)

Table S2. Pipeline stage breakdown and active components of WalkSAT/SKC hardware.

Component (Description)	Phase (Shorthand)	Count / Dimension	Latency (ns)	Energy (pJ per unit of component)	Area (μm^2 per unit of component)
Forward/Backward word line driver	WL1 / WL2	2N / M	0.501	0.27	2.73
Forward/Backward Array	FA/BA	2N x M	<0.5	0.008(ON), 0.00008(OFF)	0.0123
Forward/Backward transimpedance amplifier	TIA1/TIA2	M / 2N	0.3	0.26	10.5135
Sense amplifier	SA	2M	<0.09	0.0023	8.058
Forward/Backward register array	DFF1/DFF2	2M / 2N	<0.1	0.023	1.915
Hysteresis Comparator	HC	N	0.025	0.25	3.4865

Table S3. Area and energy breakdown for circuit components of high-order HNN hardware based on 32nm technology node.

Phase (Shorthand)	Phase (Name)	Active Components
FP	Forward Pass	DFF2(2N), WL1(2N), FA, TIA1(M), SA1(2M)
BP	Backward Pass	DFF1(2M), WL2(M), BA, TIA2(2N), HC(N)

Table S4. Pipeline stage breakdown and active components of high-order HNN hardware.

	Coherent Ising Machine (CIM)[7, 11]	D-Wave 2000Q[8,10]	sparse Ising Machine (sIM)[9]		mem-SO-HNN[4, 14]	Augmented Ising Machine (AIMs)[10]	This Work	
			FPGA sIM	Nanodevice sIM			WalkSAT/SKC	HO-HNN
**Spin representation**	Coherent light	Superconducting qubits	Digital bits	CMOS-MTJ p-bit	Digital bits	Analog charge based	Digital bits	
**Coupling representation**	Coupling matrix in FPGA	Flux storage	Sparse coupling matrix in FPGA		Memristor crossbar	Custom CMOS coupling cell	Memristor crossbar	
**Connectivity**	All-to-all	Sparse	Sparse	Sparse	All-to-all	Sparse	All-to-all	
**Dynamics**	discrete-time	continuous-time	discrete-time	continuous-time	discrete-time	continuous-time	discrete-time	
**Interaction**	High order	Second order	Second order	Second order	Second order	Third order	High order	
**Frequency**	1GHz		30MHz	1GHz	500 MHz		500 MHz	
**Time-to-Solution (TTS)**	388.9us (N=100)	44ms (N=20)	*<L> = 2555.09s (N=100)	*<L> = 77.77s (N=100)	12.12ms (N=100)	73.8us (N=100)	1.8us (N=20)<br>**51.8us** (N=100)<br>***<L>=22us**(N=100)	**0.77us** (N=20)<br>113.4us (N=100)
**Power**	50W	25kW	75W	**38.7mW**	317.2mW	300mW (N=500)	**13.35mW** (N=20)<br>66.4mW (N=100)	42.4mW (N=20)<br>209mW (N=100)
**Energy-to-Solution (ETS)**	19.445mJ (N=100)	1.1kJ	191.632kJ (N=100)	3J (N=100)	3.84mJ (N=100)		**24.03nJ** (N=20)<br>**3.43uJ** (N=100)<br>***<E>=1.46uJ** (N=100)	32.6nJ (N=20)<br>23.7uJ (N=100)
**Area**	1 km fibre ring cavity	$>10m^2$ room			0.0224 mm^2	6.76 mm^2	0.0214 mm^2 (N=100)	**0.0197 mm^2** (N=100)
**Solutions per second per watt**	51.427	$9x10^{-4}$	$5.21x10^{-6}$	0.33	260.114		**$4.1x10^7$** (N=20)<br>**$3x10^5$** (N=100)	$3x10^7$ (N=20)<br>$4.2x10^4$ (N=100)
**Solutions per second per mm^2**					$3.7x10^3$	$2x10^3$	**$9x10^5$**	$4.5x10^5$

Table S5. Comparison of proposed in-memory high-degree gradient computation hardware with current state-of-the-art accelerators.

Parameter	Value	Parameter	Value
x_{min}	0.028V	m_2	**130mV
x_{max}	1.0V	A_{V2}	4.66
v_{b1}	0.1V	v_{b2}	-0.93V
R_1	10kΩ	I_0	#100nA
I_S	0.03fA	V_{t0}	2.5V
m_1	*27.88mV	$V^{th,min}$	2.0V
A_{V1}	2	$V^{th,max}$	##3.5V
G_{on}	100μS	σ_{Gon}	$3μS
G_{off}	1μS	σ_{Goff}	$0.25μS
R_f	23.5kΩ	Δv_{th}	^^5.2mV

*$m_1=\eta(k_BT/q)$, where η =1.07 and k_BT/q = 26 mV. **$m_2=\eta(k_BT/q)$, where η=5[15] and k_BT/q = 26 mV. #I_0 is set to 100nA which corresponds to the highest analog state within in the subthreshold regime of eFlash cells in Ref. 15. ##$V^{th,max}$ is set to 3.5V which is the highest threshold voltage characterized in Ref. 15. $Standard deviations in memristor tuning is the same as that used in Supplementary Note 6. ^^ measure of flash threshold voltage tuning precision and corresponds to roughly 2% precision. Threshold voltages are sampled from a uniform distribution with bounds [v_{th}-Δv_{th} , v_{th}+ Δv_{th}], where v_{th} is the desired threshold voltage.

Table S6. Values of parameters used in simulations for real-valued gradient computation.

Supplementary Note 1: Partial derivatives and difference quotients

The gradient of a multi-variate continuous, differentiable function $H: R^N \rightarrow R$ is given by equation (1) where H_{xi} is the partial derivative of H with respect to variable x_i and is evaluated as shown in equation (2).

$$\nabla H = [\frac{\partial H}{\partial x_1}, \frac{\partial H}{\partial x_2}, \ldots, \frac{\partial H}{\partial x_N}]^T = [H_{x1}, H_{x2}, \ldots, H_{xN}] \quad (1)$$

$$H_{x1} = \lim_{h \to 0} \frac{H(x_1+h, x_2, \ldots, x_N) - H(x_1, x_2, \ldots, x_N)}{h} \quad (2)$$

The partial derivative H_{xi} tells the sign and the rate at which the function H will change when variable x_i is incremented or decremented by a certain amount. This guides gradient descent/ascent-based algorithms to iteratively update the respective variables with correct update direction/magnitude to decrease/increase the value of H. Consider a degree one bi-variate polynomial function $H = 2x_1 - 3x_2$, where $H_{x1} = 2$ and $H_{x2} = -3$. This implies that when variable x_1 is increased the function H increases with a rate of 2 whereas when variable x_2 is increased the function decreases with a rate of 3.

On the other hand, for discrete-valued functions like $H: \{a, a+h, \ldots, a+Kh\}^N \rightarrow R$ (where $\{a, a+h, \ldots, a+Kh\}$ is the discrete domain of the function with a, K and h being integers), the usual definitions of gradients and partial derivatives are not useful. For such functions, the analogue to derivative is the partial difference quotient (see Ref. 1) which is given by equation (3). Note how at the limit $h \rightarrow 0$, the difference quotient becomes the partial derivative H_{xi} in continuous space.

$$H_{x1} = \frac{\Delta H}{\Delta x_1} = \frac{H(x_1+h, x_2, \ldots, x_N) - H(x_1, x_2, \ldots, x_N)}{h} \quad (3)$$

Now for a binary valued function $H: \{0,1\}^N \rightarrow R$, the domain of each variable contains 0 and 1. So when $x_i = 0$, its only valid move is to change to 1 and vice versa. The difference quotients of H for $x_1 = 0$ and $x_1 = 1$ are shown in equation (4) and (5) (non-participating variables have been omitted for simplicity). Note that they are equal.

$$H_{x1}|_{x_1=0} = \frac{\Delta H}{\Delta x_1}|_{x_1=0} = \frac{H(x_1=1) - H(x_1=0)}{1-0} = H(x_1 = 1) - H(x_1 = 0) \quad (4)$$

$$H_{x1}|_{x_1=1} = \frac{\Delta H}{\Delta x_i}|_{x_1=1} = \frac{H(x_1=0) - H(x_1=1)}{0-1} = H(x_1 = 1) - H(x_1 = 0) \quad (5)$$

In the context of gradient descent inspired local search algorithms for solving combinatorial optimization problems that have such objective functions, the difference quotient provides information about the magnitude but not the sign/direction in which the function H will change if a variable undergoes a valid move. What is more important for such algorithms is the net change in function H when a variable x_i changes from 0 to 1 or from 1 to 0, where both the magnitude and the sign of the change are used to decide whether to accept that move or not. This is simply obtained by taking the product of the difference quotient with the difference in the final and initial variable values and is shown in equation (6).

$$\Delta H_{xi} = H_{xi} \Delta x_i \quad (6)$$

Throughout the remainder of the supplementary and main text, we shall refer to ΔH_{xi} in the context of binary valued functions H, as the "pseudo derivative of H *w.r.t.* x_i" or simply the "pseudo partial derivative". The pseudo derivative H_{xi} is the net change in the function H when binary variable x_i is flipped, i.e., when it changes its value to $\overline{x_i}$. The vector of pseudo derivatives of a multivariate function H with respect to all its variables is referred to as the "pseudo gradient".

Supplementary Note 2: Gradient computation in high-order polynomials

Any K-order discrete multi-variate polynomial in variables $\{x_1, x_2, ..., x_N\} \in X$, can be expressed as a summation of monomials (terms) - see equation (7), where t_j is the jth monomial and $t_j \in T$, the set of all monomials of H. Each of these monomials is a product of at least one variable (H might also include a constant offset term, but it is never relevant to computing derivatives, and is removed from T) and a real factor a_j. Further, let E be the set of all pairs $\{x_i, t_j\}$ such that degree of x_i in monomial t_j is non-zero (see equation (7)).

$$H = \sum_{j=1}^{M} t_j \ , \qquad t_j = a_j \prod_{i \,\forall\, \{x_i,t_j\} \in E}^{N} x_i \tag{7}$$

For such a polynomial, the pseudo derivative with respect to variable x_f (net change in the polynomial on flipping variable x_f, *i.e.,* changing value from “pre” state 0 to “post” state 1 or vice versa) is given by equation (8). The variable x_f is factored out and the rest is a sum of partial monomials. The variable x_f is factored out and the rest is a sum of partial monomials, with degree one less (in this case, removing x_f) than their respective original monomials.

$$\Delta H_{xf} = \sum_{j \,\forall\, \{x_f,t_j\} \in E} \Delta t_j \;=\; \sum_{j \,\forall\, \{x_f,t_j\} \in E} \left(t_j^{\text{post}} - t_j^{\text{pre}}\right) \;=\; \Delta x_f \sum_{j \,\forall\, \{x_f,t_j\} \in E} a_j \prod_{\substack{i \,\forall\, \{x_i,t_j\} \in E, \\ i \neq f}}^{N} x_i \tag{8}$$

Since the variables are binary, two cases arise — Case I: $x_f^{\text{pre}} = 1$ ($x_f^{\text{post}} = 0$), where initial value of x_f is 1 and Case II: $x_f^{\text{pre}} = 0$ ($x_f^{\text{post}} = 1$), where initial value of x_f is 0.

Case I ($x_f^{\text{pre}} = 1$):

The pseudo partial derivative with respect to the now-zero-valued variable is computed as:

$$\Delta H_{xf} = \sum_{j \,\forall\, \{x_f,t_j\} \in E} \left(t_j^{\text{post}} - t_j^{\text{pre}}\right) \;=\; \sum_{j \,\forall\, \{x_f,t_j\} \in E} -t_j^{\text{pre}} \;=\; -\sum_{j \,\forall\, \{x_f,t_j\} \in E} a_j \prod_{i \,\forall\, \{x_i,t_j\} \in E}^{N} x_i \tag{9}$$

We define ancillary variable $z_j = \prod_{i \,\forall\, \{x_i,t_j\} \in E} x_i$ (local to monomial t_j and consistent with the z introduced in Fig. 1e of main text) and equal to the product of the variables in that term. Observing that the product of several binary variables is one if and only if each multiplicand is one, and that the order of the term is equal to the number of multiplicands, the variables z_j can also be computed as in equation (10), where K_j is the order of the term. This replaces a product with a linear transformation, which allows for its massively parallel in-memory computation. We refer to this variable as the “break monomial indicator” as it indicates if the monomial’s value would change from non-zero to zero on flipping of any of its member variables.

$$z_j = \begin{cases} 1 \ \ if \sum_{i \,\forall\, \{x_i,t_j\} \in E} x_i = K_j \\ 0 \qquad\qquad \text{otherwise} \end{cases} \tag{10}$$

We define $\boldsymbol{B}$ as the M x N adjacency matrix of the graph G, where $\boldsymbol{B} = (\hat{\boldsymbol{b}}_1, \hat{\boldsymbol{b}}_2, ..., \hat{\boldsymbol{b}}_N)$ such that $\hat{\boldsymbol{b}}_i = (b_{1i}, b_{2i}, ..., b_{Mi})^T$ and $b_{ji} = 1$ if $\{x_i, t_j\} \in E$ and 0 otherwise. A vector-matrix multiplication between this adjacency matrix and the variables can then be used to compute the sum of variables in each term (see equation (11) below), where $\hat{\sum}_{\boldsymbol{j}}^{\textbf{mono}} = (\sum_1^{\text{mono}}, \sum_2^{\text{mono}}, ..., \sum_M^{\text{mono}})$ is the vector representing the sum of variables in each monomial (referred to as the monomial variable sum in Fig. 1c) and computed in the forward pass (see Fig 1c of main text) and $\hat{\boldsymbol{x}} = (x_1, x_2, ..., x_N)$ is the variable vector. The expression for z_j can then be re-written as shown in equation (12).

$$\hat{\sum}_{\boldsymbol{j}}^{\textbf{mono}} = \hat{\boldsymbol{x}}\, \boldsymbol{B}^{\boldsymbol{T}} \tag{11}$$

$$z_j = \begin{cases} 1 \quad if \sum_j^{\text{mono}} = K_j \\ 0 \qquad \text{otherwise} \end{cases} \tag{12}$$

Substituting the break-monomial indicator in the pseudo derivative expression (of equation (9)) and considering the monomial factors, one observes equation (13) where $\sum_{\text{f}}^{\text{break}}$ is referred to as the “break variable sum” with respect to

variable x_f. It is the number of monomials weighted by their respective factors that will become zero from non-zero when a one-valued variable x_f is flipped to zero.

$$\Delta H_{xf} = -\sum_f^{\text{break}} = -\sum_{j=1}^{M} a_j z_j b_{jf} \tag{13}$$

Case II ($x_f^{\text{pre}} = 0$) :

The pseudo partial derivative with respect to the previously-zero-valued variable is computed as shown in equation (14).

$$\Delta H_{xf} = \sum_{j\,\forall\,\{x_f,t_j\}\in E}(t_j^{\text{post}} - t_j^{\text{pre}}) = \sum_{j\,\forall\,\{x_f,t_j\}\in E} t_j^{\text{post}} = x_f^{\text{post}} \sum_{j\,\forall\,\{x_f,t_j\}\in E} a_j \prod_{\substack{i\,\forall\,\{x_i,t_j\}\in E,\\ i\neq f}}^{N} x_i \tag{14}$$

Similarly, when $x_f^{\text{pre}} = 0$ ($x_s^{\text{post}} = 1$), the monomial values pre-variable-flipping (t_j^{pre}) are equal to zero. Since $x_f^{\text{post}} = 1$, it can be factored out and set to 1. Note that since $x_f^{\text{pre}} = 0$, the product in the right-most side of equation (14) is equal to one only when all but one variable (x_f) in the monomial t_j have a value of 1. To evaluate this condition using linear transformation, we define a second ancillary variable s_j (local to term t_j) (see equation (15)) which is 1 when the sum of variables in the monomial is exactly equal to one less than the order of that term (K_j) and 0 otherwise. We refer to this variable as the "make monomial indicator" (see Fig. 1d of main text) as it indicates if the monomial's value will change from zero to non-zero on flipping of one of its member variables. The pseudo derivative for the $x_f^{\text{pre}} = 0$ case, can then be succinctly written down as in equation (16), where $\sum_f^{\text{make}}$ is the make variable sum with respect to variable x_f. It is the number of monomials weighted by their respective factors that will become non-zero from zero when a zero-valued variable x_f is flipped to one.

$$s_j = \begin{cases} 1 & if\ \sum_j^{\text{mono}} = K_j - 1 \\ 0 & otherwise \end{cases} \tag{15}$$

$$\Delta H_{xf} = \sum_f^{\text{make}} = \sum_{j\,\forall\,\{x_f,t_j\}\in E} a_j \prod_{\substack{i\,\forall\,\{x_i,t_j\}\in E,\\ i\neq f}}^{N} x_i = \sum_{j=1}^{M} a_j S_j b_{jf} \tag{16}$$

Since the pseudo derivative with respect to one-valued variables is given by the break-variable sum (equation (13)) and that with respect to zero-valued variables is given by the make-variable sum (equation (16)), both can be combined as shown in equation (17) to provide a generalized expression for the pseudo derivative of all the variables, that is equal to the difference between the make and break values.

$$\Delta\widehat{\boldsymbol{H}} = (\boldsymbol{1} - \widehat{\boldsymbol{x}}) \circ \widehat{\sum}^{\text{make}} - \widehat{\boldsymbol{x}} \circ \widehat{\sum}^{\text{break}} = ((\widehat{\boldsymbol{a}} \circ \widehat{\boldsymbol{s}})\boldsymbol{B}) \circ (\boldsymbol{1} - \widehat{\boldsymbol{x}}) - ((\widehat{\boldsymbol{a}} \circ \widehat{\boldsymbol{z}})\boldsymbol{B}) \circ \widehat{\boldsymbol{x}} \tag{17}$$

Supplementary Note 3: Converting CNF to high order polynomial.

A CNF formula comprises of conjunction of clauses (from a set $C=\{c_1, c_2, .., c_M\}$ of M clauses) where a clause is a disjunction of literals (from a set $L=\{l_1, l_2,\ldots, l_{2N-1}, l_{2N},\}$ s.t, $l_{2i-1} = x_i$ (positive literal of variable x_i), $l_{2i} = \bar{x}_i$ (negated literal of variable x_i) of $2N$ literals arising out of N variables) or in other words product of sums (AND of ORs). For example, there are two clauses in the CNF shown in Fig. 2a. The first clause has four literals and the second has two. One way of expressing the energy function of a generic CNF problem is shown in equation (18), where w_j is the weight of the clause and U_j is the indicator variable that tracks satisfaction of clause j. The merit function H then denotes the weighted sum of unsatisfied clauses, and the objective is to minimize it.

$$H = \sum_{j=1}^{M} w_j U_j \ , \quad U_j = \begin{cases} 0 \text{ if } c_j \text{ is satisfied} \\ 1 \text{ if } c_j \text{ is unsatisfied} \end{cases} \tag{18}$$

The variable U_j of each clause can be expressed as a product of linear functions of its member variables as shown in equation (19). For each of its member variable x, if its positive literal (l_{2i-1}) is present then the linear function is $(1-x)$, and if its negated literal (l_{2i}) is present then the linear function is x.

$$U_j = \prod_{i\,\forall\,\{l_{2i-1},c_j\}\in E\ or\ \{l_{2i},c_j\}\in E}^{K} g(x_i, c_j)\ , \quad \text{where } g(x_i,c_j) = \begin{cases} x_i & \text{if } \{l_{2i}, c_j\} \in E \\ 1 - x_i & \text{if } \{l_{2i-1}, c_j\} \in E \end{cases} \tag{19}$$

Take for example the CNF considered in equation (20), the energy function can then be expanded as in equation (21) & (22):

$$\text{CNF} = (\bar{x}_1||x_2||\bar{x}_3)\ \&\ (\bar{x}_2||x_3||x_4)\ \&\ (x_3||x_1||\bar{x}_4) \tag{20}$$

$$H = x_1(1-x_2)x_3 + x_2(1-x_3)(1-x_4) + (1-x_3)(1-x_1)x_4 \tag{21}$$

$$H = x_1x_3 - x_1x_2x_3 + x_2 - x_2x_3 - x_2x_4 + x_2x_3x_4 + x_4 - x_3x_4 - x_1x_4 + x_1x_3x_4 \tag{22}$$

Note how three clauses result in 10 terms in the resulting higher order polynomial. Such a conversion can lead to O(2^k) many terms in the polynomial arising out of each clause (with clause size k) in the original CNF in the worst case.

<u>Supplementary Note 4</u>: Gradient computation in Conjunctive-Normal Form

Any Constraint Satisfaction Problem (CSP) in CNF can be represented by a bipartite graph $G \in \{L,C,E\}$, where one set of vertices L denote the literals $\{l_1, l_2,\ldots, l_{2N-1}, l_{2N},\}$ s.t, $l_{2i-1} = x_i$ (positive literal of variable x_i), $l_{2i} = \bar{x}_i$ (negated literal of variable x_i), the other set C the clauses $\{c_1, c_2, .., c_M\}$ and E the set of edges defined by $\{l_i, c_j\}$ such that literal l_i is a member of clause c_j. As such, there are N variables, $2N$ literals and M clauses in the CNF. Let $\mathbf{B}$ denote the forward binary M x $2N$ adjacency matrix of G, where $\boldsymbol{B} = (\widehat{\boldsymbol{b}}_1, \widehat{\boldsymbol{b}}_2, \ldots, \widehat{\boldsymbol{b}}_{2N})$ such that $\widehat{\boldsymbol{b}}_i = (b_{1i}, b_{2i}, \ldots, b_{Mi})^T$ and $b_{ji} = 1$ if $\{l_i, c_j\} \in E$ and 0 otherwise.

The objective function in such CNF-based problems is the total number of unsatisfied clauses weighed by the clause weights w_j, where a clause is satisfied if the OR of its member literals is true and unsatisfied otherwise (see equation (23)). The goal of the optimization problem is then to minimize H. Therefore, the definition of pseudo derivatives here is the net change in the sum of weights of unsatisfied clauses when a variable is flipped. A popular score metric known as the gain is used in optimization algorithms designed for solving CSPs that are present in CNF. It is the net increase in the number of satisfied clauses weighed by their respective clause weights and can be expressed as the negative of the CNF pseudo derivative (see equation (24)).

$$H = \sum_{j=1}^{M} w_j U_j\ , \quad U_j = \begin{cases} 0 & \text{if } c_j \text{ is satisfied} \\ 1 & \text{if } c_j \text{ is unsatisfied} \end{cases} \tag{23}$$

In a CNF, when a literal is flipped not only do the clauses that contain that literal change but also the ones that contain its complement. Therefore, one needs to track the change caused by both literals associated with a given variable. The CNF gain when a variable x_f is flipped can then be expressed as the sum of gains with respect to its two literals $l_{2f\text{-}1}$ and l_{2f}.

$$\text{gain}_{x_f} = \text{gain}_{l_{2f-1}} + \text{gain}_{l_{2f}} = -\sum_{j\,\forall\,\{l_{2f-1},c_j\}\in E} w_j \Delta U_j - \sum_{j\,\forall\,\{l_{2f},c_j\}\in E} w_j \Delta U_j \tag{24}$$

Note that out of the two literals, one of them is true (one) and the other is false (zero) in any given configuration, so we evaluate the gradient contributions of the two separately. We define pl(x_f) as the literal of x_f that is currently 1 and nl(x_f) as the literal of x_f that is 0. equation (24) can then be re-written as:

$$\text{gain}_{x_f} = \text{gain}_{\text{nl}(x_f)} + \text{gain}_{\text{pl}(x_f)} \tag{25}$$

Interestingly, $\text{gain}_{\text{pl}(x_f)}$ is always a negative value and its absolute value is popularly known as the break-value (break_{xf} , analogous to but not the same as break-values that we defined in context of polynomial pseudo derivatives). This is because a clause (in CNF, a disjunction) is satisfied when any one of its literals is one. So, when a literal that is currently 1 is flipped to 0, it changes the clause status from satisfied to unsatisfied or remains the same. Therefore, break-value depicts the number of clauses that changed from satisfied to unsatisfied. Similarly, $\text{gain}_{\text{nl}(x_f)}$ is always a positive value and is known as the make-value (make_{xf}, analogous to but not the same as make-values that we defined

in context of polynomial pseudo derivatives). It depicts the number of clauses that changed from unsatisfied to satisfied. Before going ahead with the gain calculation, the linear transformation in the forward-step (a matrix-vector multiplication between the literal-vector and the adjacency matrix) that computes the sum of the literal-values in each clause is defined in equation (26).

$$\widehat{\sum}^{\boldsymbol{clause}} = \hat{\boldsymbol{l}}\, \boldsymbol{B}^{\boldsymbol{T}} \tag{26}$$

Case I (for the zero-valued literal, nl(x_f)):

The gain with respect to the zero-valued literal nl(x_f) can be written as shown in equation (27).

$$\text{gain}_{\text{nl}(x_f)} = -\sum_{j\,\forall\,\{\text{nl}(x_f),c_j\}\in E} w_j \Delta U_j \;=\; -\sum_{j\,\forall\,\{\text{nl}(x_f),c_j\}\in E} w_j (U_j^{\text{post}} - U_j^{\text{pre}}) \tag{27}$$

The variable U_j can be substituted with the Kronecker-delta function as in equation (28). U_j equates to one when the sum of its member variables is zero (clause is unsatisfied) and equates to zero when the sum of its member variables is non-zero (clause is satisfied).

$$U_j = \delta[\sum_{i\,\forall\,\{l_i,c_j\}\in E} l_i] = \delta[\sum_j^{\text{clause}}] \tag{28}$$

Since, equation (27) evaluates the CNF gain for literals that are currently 0, all clauses containing that literal would become satisfied post-flipping i.e., $U_j^{\text{post}} = 0$ (see equation (29)).

$$\text{gain}_{\text{nl}(x_f)} = -\sum_{j\,\forall\,\{\text{nl}(x_f),c_j\}\in E} w_j (0 - U_j^{\text{pre}}) \;=\; \sum_{j\,\forall\,\{\text{nl}(x_f),c_j\}\in E} w_j \delta[\sum_j^{\text{clause}}]) \tag{29}$$

A clause-local variable s_j (also referred to as the make-clause indicator in Fig. 2d) as defined in equation (30) can then be substituted in equation (29) to yield the following expression for the gain as shown in equation (31). Here $\sum_j w_j b_{j,\text{nl}(x_f)} s_j$ is the output that is computed in the backward make pass VMM (see Fig. 2d) and is referred to as the make-literal sum (see equation (32)) of literal nl(x_f). Since nl(x_f) is the zero-valued literal, its make-literal sum is also the make-value of variable x_f. The make-value of a variable is therefore the make literal sum (backward make pass output) of its zero-valued literal. To generalize the expression in equation (31) and write it without exact information about the zero-valued literal, we write the make-value of x_f as the sum of make-literal sums scaled by the inverted literal value of both its literals as shown in equation (33).

$$s_j = \begin{cases} 1 & \text{if} \sum_j^{\text{clause}} = 0 \\ 0 & \text{otherwise} \end{cases} \tag{30}$$

$$\text{gain}_{\text{nl}(x_f)} = \sum_{j\,\forall\,\{\text{nl}(x_f),c_j\}\in E} w_j s_j \;=\; \sum_{j=1}^{M} w_j b_{j,\text{nl}(x_f)} s_j \tag{31}$$

$$\sum_{\text{nl}(x_f)}^{\text{make}} = \sum_{j=1}^{M} w_j b_{j,\text{nl}(x_f)} s_j \tag{32}$$

$$\text{gain}_{\text{nl}(x_f)} = (1 - l_{2f-1})\sum_{l_{2f-1}}^{\text{make}} + (1 - l_{2f})\sum_{l_{2f}}^{\text{make}} \;=\; (1 - l_{2f-1})\sum_{j=1}^{M} w_j b_{j,2f-1} s_j + (1 - l_{2f})\sum_{j=1}^{M} w_j b_{j,2f} s_j \tag{33}$$

Case II (for the one-valued literal, pl(x_f)):

The gain with respect to the one-valued literal pl(x_f) can be written as shown in equation (34).

$$\text{gain}_{\text{pl}(x_f)} = -\sum_{j\,\forall\,\{\text{pl}(x_f),c_j\}\in E} w_j \big(U_j^{\text{post}} - U_j^{\text{pre}}\big) \;=\; -\sum_{j\,\forall\,\{\text{pl}(x_f),c_j\}\in E} w_j (U_j^{\text{post}} - 0) \tag{34}$$

Since we are considering the gain for pl(x_f) (the literal that is currently one), U_j^{pre} can be set to 0 in equation (34), since clause j is satisfied. Subsequently, U_j^{post} can be replaced with $\delta[\sum_j^{\text{clause}}\text{-}1]$, where $\sum_j^{\text{clause}}$ denotes the sum of literals pre-flipping and the '1' is subtracted from it to reflect the literal pl(x_f) changing from 1 to 0.

$$\text{gain}_{\text{pl}(x_f)} = -\sum_{j\,\forall\,\{\text{pl}(x_f),c_j\}\in E} w_j \delta[\sum_j^{\text{clause}} - 1] \tag{35}$$

Additionally, we define another clause-local variable z_j known as the break-clause indicator (see Fig. 2e) as shown in equation (36).

$$z_j = \begin{cases} 1 & \text{if } \sum_j^{\text{clause}} - 1 = 0 \\ 0 & \text{otherwise} \end{cases} = \begin{cases} 1 & \text{if } \sum_j^{\text{clause}} = 1 \\ 0 & \text{otherwise} \end{cases} \tag{36}$$

Substituting this new variable in the gain expression, the final gain can then be written as shown in equation (37), where $\sum_{j=1}^{M} w_j b_{j,\text{pl}(x_f)} z_j$ is the output that is computed in the backward break pass VMM and is known as the break-literal sum of literal pl(x_f) denoted by $\sum_{\text{pl}(x_f)}^{\text{break}}$ (see Fig. 2e).

$$\text{gain}_{\text{pl}(x_f)} = -\sum_{j\,\forall\,\{\text{pl}(x_f),c_j\}\in E} w_j z_j \;=\; -\sum_{j=1}^{M} w_j b_{j,\text{pl}(x_f)} z_j \;=\; -\sum_{\text{pl}(x_f)}^{\text{break}} \tag{37}$$

Since pl(x_f) is the one-valued literal, its break-literal sum is also the break-value of variable x_f. The break-value of a variable is therefore the break literal sum (backward break pass output) of its one-valued literal. To generalize the expression in equation (37) and write it without exact information about the one-valued literal, we write the gain of pl(x_f) as the sum of break-literal sums scaled by the non-inverted literal value of both literals of x_f as shown in equation (38). The absolute value of the expression in equation (38) is the break-value of variable x_f.

$$\text{gain}_{\text{pl}(x_f)} = -l_{2f-1}\sum_{l_{2f-1}}^{\text{break}} - l_{2f}\sum_{l_{2f}}^{\text{break}} \;=\; -l_{2f-1}\sum_{j=1}^{M} w_j b_{j,2f-1} z_j - l_{2f}\sum_{j=1}^{M} w_j b_{j,2f} z_j \tag{38}$$

The net gain of the variable is the sum of gains associated with its positive and negated literals or in other words the difference between make and break-values (see equation (25)) and therefore it can be written as shown in equation (39) & (40). The clause-weight vector $\widehat{\boldsymbol{w}}$ is unitary for K-SAT problems.

$$\text{gain}_{x_f} = \{(1-l_{2f-1})\sum_{l_{2f-1}}^{\text{make}} + (1-l_{2f})\sum_{l_{2f}}^{\text{make}}\} - \{l_{2f-1}\sum_{l_{2f-1}}^{\text{break}} + l_{2f}\sum_{l_{2f}}^{\text{break}}\} \tag{39}$$

$$\text{gain}_{x_f} = \{(1-l_{2f-1})(\widehat{\boldsymbol{w}}\circ\widehat{\boldsymbol{s}})\,.\,\widehat{\boldsymbol{b}}_{2f-1} + (1-l_{2f})(\widehat{\boldsymbol{w}}\circ\widehat{\boldsymbol{s}})\,.\,\widehat{\boldsymbol{b}}_{2f}\} - \{l_{2f-1}(\widehat{\boldsymbol{w}}\circ\widehat{\boldsymbol{z}})\,.\,\widehat{\boldsymbol{b}}_{2f-1} + l_{2f}(\widehat{\boldsymbol{w}}\circ\widehat{\boldsymbol{z}})\,.\,\widehat{\boldsymbol{b}}_{2f}\} \tag{40}$$

<u>Supplementary Note 5</u>: Single-step backward pass with three-terminal crossbar arrays.

When passive cross point devices (like 0T1R memristors) are replaced with three-terminal devices (like 1T1R memristor bit-cells), the backward step can be executed in the same crossbar array in a single cycle, instead of using two separate arrays like shown in Fig. S4e. To demonstrate this, consider the gradient computation in polynomial forms and CNF separately as described below.

Case A (Polynomials):

Using equation (17), one can write down the gradient with respect to a single variable x_f as follows (we assume all monomial factors to be one for simplicity):

$$\Delta H_{xf} = (1-x_f)\sum_f^{\text{make}} - x_f\sum_f^{\text{break}} \;=\; \widehat{\boldsymbol{s}}.\widehat{\boldsymbol{b}}_{\boldsymbol{f}}(1-x_f) - \widehat{\boldsymbol{z}}.\widehat{\boldsymbol{b}}_{\boldsymbol{f}} x_f \;=\; (-\widehat{\boldsymbol{z}}\,x_f + \widehat{\boldsymbol{s}} - \widehat{\boldsymbol{s}}x_f).\widehat{\boldsymbol{b}}_{\boldsymbol{f}} \tag{41}$$

Note that we can rewrite $\widehat{\boldsymbol{s}} = \widehat{\boldsymbol{s}}\circ\widehat{\boldsymbol{s}}$ (as elementwise product with itself) without loss of generality since it is a binary-valued vector. Also, based on definitions of $\widehat{\boldsymbol{s}}$ and $\widehat{\boldsymbol{z}}$ (in equation (12) and (15)), one can see that both vectors cannot have their entries at the same position to be equal to one. This means s_j x z_j is always equal to zero. This allows us to re-write equation (41) as shown below:

$$\Delta H_{xf} = (\widehat{\boldsymbol{s}}\circ\widehat{\boldsymbol{z}} - \widehat{\boldsymbol{z}}\,x_f + \widehat{\boldsymbol{s}}\circ\widehat{\boldsymbol{s}} - \widehat{\boldsymbol{s}}\,x_f).\widehat{\boldsymbol{b}}_f \;=\; \big((\widehat{\boldsymbol{s}}+\widehat{\boldsymbol{z}})\circ(\widehat{\boldsymbol{s}}-x_f)\big).\widehat{\boldsymbol{b}}_f \tag{42}$$

The same equation can then be expanded using a summation as seen below:

$$\Delta H(x_f) = \sum_{j=1}^{M}(s_j + z_j)(s_j - x_f)\,b_{fj} \tag{43}$$

The expression inside the summation of equation (43) denotes a triple product and is identical to the expression (equation (44)) for current flowing in a bit line of a 1T1R crossbar array (see Fig. S6).

$$I_f = \sum_{j=1}^{M} \theta(V_{gj} - V_{\text{th}})(V_{Cj} - V_{Xf})\, G_{jf} \quad (44)$$

Since the signal s_j+z_j is binary ("+" denoting arithmetic addition here, not logical disjunction, as s_j and z_j cannot be unity at the same given time) it acts as the gating signal gate$_j$. Depending on whether gate$_j$ is 0 or 1, the voltage V_{gj} applied to the gate of the transistor in Fig. S6 is chosen to turn the switch off or on respectively ($\theta(V_{gj} - V_{\text{th}}) = 1$ when $V_{gj} \geq V_{\text{th}}$ and $= 0$ when $V_{gj} < V_{\text{th}}$). Voltage V_{Cj} proportional to s_j (=s_jV_0) is applied to the other terminal of the transistor from the monomial side (along the rows) and V_{xi} proportional to variable value x_f (=$x_f V_0$) is applied along the column lines from the variable side. The normalization factor between equations (43) and (44) is the unit current via on-state coupling weight, $I_0 \equiv V_0 G_{\text{on}}$.

Case B (CNFs):

One can compactly re-write equation (40) as shown in equation (45) (we assume all clause weights to be one).

$$\text{gain}_{x_f} = \sum_{k=0}^{1} \left(\hat{\boldsymbol{s}}(1 - l_{2f-k}) - \hat{\boldsymbol{z}} l_{2f-k}\right) . \hat{\boldsymbol{b}}_{2f-k} \quad (45)$$

After doing manipulations like what was introduced in equation (42) (in Case A), we get equation (46).

$$\text{gain}_{x_f} = \sum_{k=0}^{1} \left(\hat{\boldsymbol{s}} - \hat{\boldsymbol{s}} l_{2f-k} - \hat{\boldsymbol{z}} l_{2f-k}\right) . \hat{\boldsymbol{b}}_{2f-k} = \sum_{k=0}^{1} \left(\hat{\boldsymbol{s}} \circ \hat{\boldsymbol{z}} + \hat{\boldsymbol{s}} \circ \hat{\boldsymbol{s}} - \hat{\boldsymbol{s}} l_{2f-k} - \hat{\boldsymbol{z}} l_{2f-k}\right) . \hat{\boldsymbol{b}}_{2f-k} = \sum_{k=0}^{1} (\hat{\boldsymbol{s}} + \hat{\boldsymbol{z}}) \circ (\hat{\boldsymbol{s}} - l_{2f-k}) . \hat{\boldsymbol{b}}_{2f-k} \quad (46)$$

Re-writing the same in the summation form we get equation (47)-(48).

$$\text{gain}_{x_f} = \sum_{j=1}^{M} \sum_{k=0}^{1} (s_j + z_j)(s_j - l_{2f-k})\, b_{j,2f-k} \quad (47)$$

$$\text{gain}_{x_f} = \sum_{j=1}^{M} (s_j + z_j)(s_j - x_f) b_{j,2f-1} + \sum_{j=1}^{M} (s_j + z_j)(s_j - \bar{x}_f) b_{j,2f} \quad (48)$$

$$I_{xf}^{\text{total}} = I_{xf} + I'_{xf} = \sum_{j=1}^{M} \theta(V_{gj} - V_{th})(V_{cj} - V_{xf}) G_{j,2f-1} + \sum_{j=1}^{M} \theta(V_{gj} - V_{th})(V_{cj} - V'_{xf}) G_{j,2f} \quad (49)$$

Like Case A, the two inner summations expressed in equation (47-48) can be mapped to the current flowing through a pair of bit-lines (corresponding to the two literals of a variable) of a 1T1R crossbar array that is summed at the periphery as shown in equation (49) (see Fig. S10). Depending on whether the binary-valued signal gate$_j$ = s_j+z_j is 0 or 1, the transistor gate voltage V_{gj} ((see Fig. S10c)) is set such that it turns the transistors in that row off or on respectively ($\theta(V_{gj} - V_{\text{th}}) = 1$ when $V_{gj} \geq V_{\text{th}}$ and $= 0$ when $V_{gj} < V_{\text{th}}$). The other set of voltages V_{cj} applied from the clause side (along the rows in Fig. S10) is proportional to the make clause indicator signal s_j (=s_jV_0), while the voltages V_{xf} and V'_{xf} applied along the columns (in Fig. S10) from the literal side are proportional to the literal values x_f (=$x_f V_0$) and $\bar{x}_f$ (=$\bar{x}_f V_0$) respectively. The $b_{j,2f-k}$ values are mapped to the conductance $G_{j,2f-k}$ of the crosspoint devices. The normalization factor between equation (48) and (49) is the unit current via on-state coupling weight, $I_0 \equiv V_0 G_{\text{on}}$.

<u>Supplementary Note 6</u>: SPICE simulation of dot-product error based on crossbar noise.

SPICE simulations of a vast ensemble of analog computations using 1T1R crossbars, were performed to study the impact of varying maximum order of mapped K-SAT problem, clause-to-variable ratio and problem size and crossbar dimensions on the accuracy of computed clause currents, break and gain values. The transistor in the 1T1R device was emulated using a linear resistor having resistance 950Ω or 1GΩ depending on whether the gate signal was turned ON or OFF respectively, for faster simulation convergence. Memristors were emulated using resistors with conductance values drawn from normal distribution with mean set to G_{on} = 100µS and G_{off} = 1µS and standard deviations 3uS and 0.25uS depending on whether 1 and 0 are stored respectively, to reflect memristor programming errors. The standard deviation values were chosen based on experimentally realized programming errors at those conductance levels in Ref. 2. Line resistance of 1Ω per crosspoint device was considered for emulating bit-line and word-line resistances. The forward and backward crossbars were biased as described in Fig. S10 with the read voltage of memristors V_{on} being set to 0.2V.

Fig. S12 shows the error (three times the standard deviation) in clause currents in the forward array when K-SAT problems of different maximum order K are mapped to the crossbar and histograms for specific values of K. Uniform random SAT problems with 14 variables, 64 clauses and K = 3, 4, 5, 6, 7, 8, 9, 10 were generated as described in the Methods section and were mapped to crossbar of dimension 64 x 64. Literal values corresponding to 500 randomly generated variable assignments were applied as inputs to the crossbar (see Fig. S10). The number of valid clause current levels increase with K and so does the error at each level. However, since the errors in $\sum^{\text{clause}} = 0$ and $\sum^{\text{clause}} = 1$ and their overlap region (as well as overlap of $\sum^{\text{clause}} = 1$ and $\sum^{\text{clause}} = 2$) is not significantly degraded, make and break clause indicator signals (determined by whether clause current is equal to zero and one respectively) can be reliably computed even as K increases for the considered problem size. Fig. S13 shows the errors in measured break and gain values for problems with different maximum order and histograms for specific values of K. Error in make values is expected to follow similar trend as break values as both are subjected to equivalent underlying hardware conditions. Interestingly, the number of valid levels (in break and gain currents) as well as their error decreases with increasing K. The magnitudes of both these currents depend on the make and break literal sums ($\sum^{\text{make}}$ and $\sum^{\text{break}}$) which in turn are proportional to the number of clauses with make and break indicator signals (s_j and z_j) equal to one. Statistically, the probability of a clause having $s_j = 1$ is ~ $1/2^K$ and $z_j = 1$ is ~ $K/2^K$. Therefore, the number of clauses that contribute to the break and gain currents decrease with increasing K and hence the decrease in the number of their valid levels. This also results in reduced errors as there are fewer leakage currents flowing in the bit lines (contributed by OFF state crosspoint devices with activated word lines and equal to $V_{\text{on}}G_{\text{off}}$). Another parameter that changes with different high order optimization problems is the clause-to-variable ratio (M/N). Fig. S14 shows the errors in measured break and gain values for problems with different M/N values and histograms for specific values of M/N. Uniform random SAT problems with 64 clauses, K = 3 and varying number of variables were generated as described in the Methods section and were mapped to crossbar of dimension 64×64. Increasing clause-to-variable ratio does not seem to have a significant effect on the number of valid levels or error.

Although all problems considered till now could be mapped to 64×64 crossbar arrays, larger problems would inevitably require larger arrays. Increasing crossbar dimensions leads to larger errors due to higher IR drops along the now longer word and bit-lines and leakage currents. Fig. S15 shows the error in clause currents in the forward array for larger problem sizes and crossbar array dimensions and histograms for specific problem sizes. As expected, errors increase with increasing crossbar array and problem size, but overall accuracy of forward operation is not degraded as the thresholds for classifying the $\sum^{\text{clause}} = 0$ and $\sum^{\text{clause}} = 1$ levels are still well defined. Fig. S16 shows the increase in break and gain value computation error in the backward operation for increasing problem and crossbar sizes. However, errors in the backward operation are on average 3-5 times larger than those in the forward operation (see Fig. S16). This is because for the considered 3-SAT problems, each clause in the forward array has at most three ON state memristors in their bit line, whereas each literal has much higher number of ON state memristors (5 to 10). This leads to higher leakage currents contributed by OFF state memristors with activated word lines ($V_{\text{on}}G_{\text{off}}$). Arrays of size 512×512 that can accommodate uniform random 3-SAT problems of up to 100 variables show much higher errors, with significant overlap between neighboring gain/break current levels (see Fig. S16 c, f).

To study the impact of memristor variation on error in gain and break value computation, we performed backward array simulations with a 128x128 memristor crossbar array corresponding to a uniform random 3-SAT problem with 20 variables and 91 clauses, tuned with different levels of errors (see Fig. S17). Both gain and break-value errors are borderline (with maximum value of its three times sigma close to 0.5) for up to 240% and 2.3% tuning precision (corresponding to $\sigma_{\text{Goff}} = 3\mu\text{S}$ and $\sigma_{\text{Gon}} = 3\mu\text{S}$) of the OFF and ON state memristors respectively.

<u>Supplementary Note 7</u>: Circuit Blocks of WalkSAT/SKC solver.

Figure. S18a shows the circuit blocks required for implementing a WalkSAT/SKC solver hardware that is enabled by in-memory clause evaluation and break-value computation. Such an implementation follows the ideas laid down in Fig. 2 of main text and Fig. S10. The forward and backward arrays are driven by $2N$ and M word-line drivers corresponding to literal and clause indicator inputs respectively. Transimpedance amplifiers are used to convert the bit line currents to voltage, that are then compared against fixed thresholds using sense amplifiers. For the forward array, there are two sense amplifiers per bit line (hence $2M$ total count for SA1s) to evaluate both make and break clause indicator signals in parallel. For the backward array, each variable has a 2-to-1 analog multiplexer to pass either

its make or break current for voltage conversion. A voltage-mode analog winner-take-all circuit (see Ref. 3) is implemented to choose the variable with the minimum break value. Register arrays (DFFs) are used to latch clause indicator signals and literal values in each cycle.

Table S1 shows the description, count/dimension, energy, latency, and area estimates for each of the circuit blocks. All estimates are based on circuit implementation in 32nm technology node. The memristors in the crossbar arrays are assumed to have G_{ON} = 100µS and G_{OFF} = 1µS, whereas the memristor cell size is assumed to be $12F^2$ (F=32nm). The read voltage (V_{ON}) applied across the memristors is assumed to be 0.2V.The estimates for the word line drivers, transimpedance amplifier and register arrays are adopted from Ref. 4, whereas those for the sense amplifier, analog multiplexer and winner take all circuits are based on SPICE simulations and physical layouts of the respective modules in 65nm technology node and then scaling down to 32nm.

Figure. S18b shows the timing diagram of the WalkSAT/SKC solver for a single thread (a thread corresponds to a unique random variable state initialization). Overall execution is divided into six pipeline stages: FP (Forward Pass), RS (Random Clause Select), BMP (Backward Make Pass), BBP (Backward Break Pass), BWP (Backward WTA Pass) and FL (Variable Select & Flip) (see Table S2), where each stage takes one clock cycle to update. In FP, the forward array evaluation takes place as a result of which the make clause indicator signals (s_j) are sent to the digital control logic and the break clause indicator signals (z_j) are sent to be latched by the register array in the next cycle. In RS stage, the forward pass results are latched and one unsatisfied clause is randomly selected in the control logic. In BMP stage, only one word line drive corresponding to the selected clause is activated. The analog multiplexer is configured to pass make currents and the backward array peripherals are used to detect the member variables of the unsatisfied clause. In BBP stage, the z_j values latched in DFF1 are used to drive the backward array and bit line peripherals are configured to compute break values of the member variables. In BWP stage, the same voltage output of the TIAs as obtained in the BBP stage is used to detect the variable with minimum break value using the winner take all circuit. Finally in FL stage, the variable is chosen by the control logic based on the WalkSAT/SKC heuristic (see Methods section) and final values (post-flipping) are latched in the register array (DFF2). Based on the latency estimates of the circuit blocks and the timing diagram, the critical path delay in any stage (excluding the WTA) is approximately 1.4ns. Assuming an additional slack of 0.6ns to achieve a reasonable dynamic range in the WTA and to account for additional overheads associated with full chip design, we set the operating frequency to 500 MHz (T_{clk} = 2ns), similar to Ref. 4.

While the backward array is operated during BMP, BBP and BWP stages, the remaining stages are idle. To increase hardware utilization, Fig. S18c shows the timing diagram for a pipelined dual-thread implementation of the solver. In this implementation, a second thread (with a different state initialization) is launched three clock cycles after the first is started. As a result, the second thread goes through the FP stage while the first goes through the BBP stage. The latency of each thread's flip is still the same (six clock cycles) but the throughput is doubled. The advantage for such multi-threaded implementations of stochastic local search solvers lies in the fact that the performances of such solvers also depend on the state at which they are initialized. Therefore, a second thread running in parallel increases the probability of converging in a given time by roughly two times (see Ref. 5).

<u>Supplementary Note 8</u>: High Order Discrete State/Time Hopfield Neural Network.

To highlight the benefits of high order full gradient computation, we considered possible hardware implementation and modeling of a version of the High-Order Hopfield Neural Network (HO-HNN) (see Methods for details on the algorithm). Unlike WalkSAT/SKC that uses only break-values, HO-HNN makes use of full gain information of all variables to update states. Moreover, in contrast to other implementations of hardware HNNs and simulated annealing that use gradient information of single or sub-groups of neurons, our implementations evaluate and uses the gain/gradient information of all neurons in parallel to update neuron states. Such massively parallel gain computation is possible using our proposed hardware paradigm comprising of a forward and backward array.

Fig. S19a shows the circuit blocks required for implementing a HO-HNN hardware that is enabled by in-memory gain computation. Such an implementation follows the ideas laid down in Fig. 2 of main text and Fig. S10 for solving high order combinatorial optimization problems encoded in CNF. For problems whose cost function is natively present in the polynomial form, a similar hardware can be used with trivial modifications, where clauses and literals are replaced by monomials and variables respectively (see Fig. 1 of main text and Supplementary Note 5 for further details). Several

hardware blocks are similar to those in Fig. S18a. Transimpedance amplifiers are used to perform current to voltage conversion of the clause currents in the forward array and the net gain current currents in the backward array. In the backward array, Hysteretic Comparators (HC)[4] are used instead of sense amplifiers, to compare the gain value against a noisy threshold and guide evolution of the neuron states.

Table S3 shows the description, count/dimension, energy, latency, and area estimates for each of the circuit blocks used in HO-HNN implementation. The estimates for the hysteretic comparators are adopted from Ref. 4. Fig. S19b shows the timing diagram of the HO-HNN solver for a single thread. Owing to the rather simpler structure of the HO-HNN algorithm, the execution is divided into just two pipeline stages: FP (Forward Pass) and BP (Backward Pass) (see Table S4), where each stage takes one clock cycle to update. In FP, the forward array evaluation takes place and resultantly the make and break clause indicator signals (s_j and z_j) are latched by the $2M$ sized register array. In BP stage, the s_j and z_j values latched in DFF1 are used to drive the backward array and bit line peripherals are used to compute and compare gain values of all variables/neurons against dynamically changing threshold values and update neuron state. Similar to the WalkSAT/SKC solver, we set the operating frequency to 500 MHz (T_{clk} = 2ns). To increase hardware utilization, Fig. S19c shows the timing diagram for a pipelined dual-thread implementation of the solver. The latency of each thread's flip is two clock cycles (same as single thread implementation) but the throughput is doubled (one flip per clock cycle). A second thread running in parallel increases the probability of converging in a given time by roughly two times.

<u>Supplementary Note 9</u>: Hardware Aware Simulations.

We performed hardware aware simulations of the two algorithms: WalkSAT/SKC and High Order HNN, by modeling the break-value/gain computation error results for different crossbar sizes and memristor programming errors. Scaling trends in time and energy to solution were obtained by solving uniform random 3-SAT instances. Specifically, 80 generated instances of 14-variable 64-clause 3-SAT problems were used (see Methods section for details on instance generation) and instances uf20-(921-1000), uf50-(921-1000), uf75-(51-100) and uf100-(921-1000) from SATLIB[6] were used for 3-SAT problems with N = 20, 50, 75 and 100 respectively. Optimal values of hyperparameters p, MAX_FLIPS for WalkSAT/SKC and r, T_0, MAX_FLIPS for HO-HNN were obtained separately for each problem size. Grid search was implemented to find the set of hyperparameters that led to minimum batch TTS (see Methods section for details). The grid search was performed on a different set of instances, specifically uf20-(901-920), uf50-(901-920), uf75-(51-60) and uf100-(901-920) for 3-SAT problems with N = 20, 50, 75 and 100 respectively. Due to similarity in number of variables, the hyperparameters for solving the 14-variable 3-SAT instances were set to the optimal hyperparameters for the 20-variable 3-SAT instances.

Fig. S20a shows the scaling trend in batch TTS with problem size for the two heuristics when ideal and realistic memristor programming errors are considered, whereas Fig. S20b shows the impact of different levels of memristor programming error on batch TTS for 20-variable SAT problems. For larger problems (N=100), there is 8% drop in batch TTS (HO-HNN heuristic) when memristors are programmed with 2.5% and 20% error margins for high and low conductance regimes respectively, compared to the case with 0% memristor programming error margins. For higher memristor programming error margins (16% and 235% for high and low conductance respectively), there is up to 6% drop in batch TTS (HO-HNN heuristic) at even smaller problem sizes with N = 20. Both heuristics seem to be robust to computation errors, potentially due to requirement of binary weights (resulting in larger margins) and the inherent navigational properties of the heuristics themselves. For example, with WalkSAT/SKC, good performance has been observed to be associated with successfully being able to flip a variable with zero break-value when present as well as the accuracy of the step where the variable with minimum break-value is flipped. Although computation error in break-values increase with larger crossbar sizes and memristor programming tolerances, the probability that a variable with break-value = 0 is interpreted as that with break-value = 1 is relatively low. Moreover, even if it does fail to detect presence of variable with break-value = 0, the minimum break-value detection step might be able to flip the correct variable (since its computed break-value is still low compared to other variables).

All data points in Fig. S20 and Fig. 5a of the main text are represented in number of variable flips, without considering any hardware aspects like cycle time, pipeline stages and single/multiple thread implementations. The TTS in # of variable flips can be converted to hardware TTS (in seconds) using equation (50), where t_{clk} is the cycle time,

speedup_factor is equal to the number of threads and # of pipeline stages is set to six and two for WalkSAT/SKC and HO-HNN respectively (see Supplementary Note 7-8).

$$\text{TTS (in seconds)} = \text{TTS(in \# of variable flips)} * t_{\text{clk}} * \frac{\text{\# of pipeline stages}}{\text{speedup\_factor}} \tag{50}$$

All data points in Fig. 5b of the main text represent hardware TTS (in seconds) with t_{clk} = 2ns and speedup_factor = 2 (corresponding to multi-threaded execution). The expression for Energy-to-Solution (ETS, in Joules) plotted in Fig. 5c of main text is shown in equation (51), where E_{avg} is the average energy (in Joules) spent per clock cycle by the respective heuristics.

$$\text{ETS (in Joules)} = \text{TTS(in \# of variable flips)} * \frac{\text{\# of pipeline stages}}{\text{speedup\_factor}} * E_{\text{avg}} \tag{51}$$

The expression for E_{avg} for WalkSAT/SKC and HO-HNN are given by equation (52) & (53), respectively.

$$E_{\text{avg}}^{\text{WalkSAT/SKC}} = \text{speedup\_factor} * \frac{E_{\text{FP}}+E_{\text{RS}}+E_{\text{BMP}}+E_{\text{BBP}}+E_{\text{BWP}}+E_{\text{FL}}}{\text{\# of pipeline stages}} \tag{52}$$

$$E_{\text{avg}}^{\text{HO-HNN}} = \text{speedup\_factor} * \frac{E_{\text{FP}}+E_{\text{BP}}}{\text{\# of pipeline stages}} \tag{53}$$

The stage-specific energy values ($E_{\text{FP}}, E_{\text{RS}}, E_{\text{BMP}}, E_{\text{BBP}}, E_{\text{BWP}}, E_{\text{FL}}$) and ($E_{\text{FP}}, E_{\text{BP}}$) for WalkSAT/SKC and HO-HNN respectively, are obtained by summing up the energy spent in the different hardware components that are active in that stage scaled by the size/count of those components (see Table S1-S2 for WalkSAT/SKC and Table S3-S4 for HO-HNN).

Supplementary Note 10: Comparison with other approaches.

In Table S5, performance of the proposed hardware is compared with other technologies in solving high-order combinatorial optimization problems, specifically the uniform random 3-SAT problem. The other technologies include the Coherent Ising Machine (CIM)[7], D-Wave 2000Q[8], sparse Ising Machines (sIM)[9], memristor crossbar based second order only HNN (mem-SO-HNN) and the Augmented Ising Machine (AIMs)[10]. All results pertaining to our hardware are based on experimentally grounded simulations (see Supplementary Note 9 and Fig. 5 of main text), whereas the results for D-Wave and FPGA-based sIM are based on experimental results. The results for CIM, MTJ p-bit based sIM, mem-SO-HNN and AIMs are based on modeling results.

For CIM, the TTS for the SAT-CFC[11] algorithm on 100-variable uniform random 3-SAT instances (in number of round trips) was obtained from Ref. 11 and multiplied with the single round trip time (~2.5N ns) of NTT-CIM system[7] to obtain the hardware TTS in seconds. A lower bound for the power consumption of the system was estimated by considering the power consumption of the FPGAs and is roughly equal to 50W (two Xilinx Virtex-7 FPGAs are used in the NTT-CIM system, see Ref. 7, 12). The ETS was obtained by taking the product of the power with TTS. For the D-Wave system, experimental TTS values were obtained from Ref. 10 for 20-variable SAT problem.

The results for the sparse Ising Machine have two parts: FPGA-based and Nanodevice-based. Results pertaining to the former are based on experimental results from FPGA, while the latter is based on modeling results where spin and random number generation circuitry is assumed to be replaced with CMOS + Magnetic Tunnel Junction (MTJ) based p-bits. Note that although Ref. 9 reports something called Time-to-Solution, it is different from it is defined in this paper. For example, TTS_{99} in Ref. 9 is the time taken to satisfy 99% of the clauses of the 3-SAT problem, whereas TTS_{99} in our paper is the time taken to *fully* solve the problem with 99% certainty. Since we do not measure time taken to satisfy a fraction of the clauses, we chose to compare TTS_{100} values from Ref. 9 (which refers to time taken to satisfy 100% of the clauses, or in other words the entire SAT instance) with the mean time to solve 3-SAT problems using our hardware. We denote it as $\langle L_i \rangle$ to distinguish with the definition of TTS used in this paper. The TTS_{100} (or $\langle L_i \rangle$) for the FPGA-sIM (with k=4 configuration and 30 MHz clock frequency) on 100-variable 3-SAT problems was directly obtained from Ref. 9. The throughput for this problem size is ~$5.5\text{x}10^{10}$ flips-per-second (fps). When nanodevices are used, the throughput increases to approximately $1.8\text{x}10^{12}$ fps (see Ref. 9). If the dynamics of the Ising Machine remain the same and therefore the number of effective flips taken to solve the problem, the $\langle L_i \rangle$ for a

Nanodevice-sIM on 100-variable SAT problem is obtained by scaling the <L_i> of the FPGA-sIM with the ratio of their throughputs. The power consumption of the FPGA-sIM is ~75W (equal to the power of the AMD Ultrascale+ FPGA used in the work, see Ref. 9, 13). The power consumption of Nanodevice-sIM is calculated by assuming 20μW per p-bit based spin, with a total of 1935 p-bits in the sparse Ising Machine.

We also consider a memristor crossbar based HNN that can implement only second-order interactions, referred to as SO-HNN. A single memristor crossbar array with dimensions equal to (N+M) x 2(N+M) is used to implement the second-order coupling matrix of the QUBO form of the 3-SAT problem, using analog memristor conductance. Factor 2 arises from the requirement of positive and negative weights. Apart from the increased crossbar size, the requirement of analog-tunable memristors is another key feature that sets such SO-HNNs apart from the HO-HNN or WalkSAT/SKC hardware proposed in this work, that only require binary memristors. The remaining peripherals of the crossbar array include the appropriate number of WL drivers, register arrays, TIAs and Hysteretic Comparators (discussed in Supplementary Note 8). Due to the similarity in hardware components, we assume an optimistic 2ns clock period to provide fair comparison with the proposed hardware, even though the crossbar sizes required by SO-HNN are larger for the same problem sizes. The TTS (in number of flips) for solving a 100-variable SAT problem is obtained from Ref. 14 (QUBO, parallel updates) and subsequently scaled with the clock period to obtain hardware TTS (in seconds). Energy per cycle for the SO-HNN hardware is calculated by adding the energy contributions of the constituent peripherals and crossbar array (using values from Table S3) and is then scaled up by the TTS (in number of flips) to obtain ETS.

The TTS of Augmented Ising Machine (AIMs) to solve 100-variable SAT problems is also considered. A crude power estimate for N=500 is provided in Ref. 10, but it is unclear how that number scales with problem size. The area for an N=500 AIMs chip is given to be 13x13 mm^2. Based on our previous analysis, the area for AIMs seems to scale with ~N^2 and therefore a rough estimate for such a system with N=100 would be 6.76 mm^2. Note that AIMs implement continuous-time dynamics, whereas our modeled hardware implements discrete-time dynamics in its current version. Despite that, the performance in terms of TTS is at par. Continuous-time systems are supposed to have much higher navigational efficiency compared to their discrete-time parts, resulting in faster TTS. The peripherals of the proposed high-degree gradient computation hardware can be modified to implement continuous-time dynamics and potentially achieve even faster TTS.

Our hardware has at least 7.7x and 1.4x faster TTS when compared to discrete (CIM) and continuous-time systems (AIMs). Even at iso-frequency with FPGA-sIM (assuming the operation frequency of our hardware is scaled down to 30 MHz), the resultant mean time to solve 100-variable problems is six orders of magnitude faster, owing to the capability of implementing all-to-all connectivity and solving 3-SAT problems in their native form using advanced heuristics like WalkSAT/SKC. It has three orders of magnitude lower ETS when compared to a memristor based SO-HNN for 100-variable SAT problems, owing to compact high-order hardware footprint and higher navigational efficiency of native high-order solvers. The proposed hardware has at least two orders of magnitude higher throughput per watt and throughput per unit area compared to all other technologies. A higher throughput per unit area indicates that at iso-area multiple replicas of the Ising Machine can be launched in parallel to attain even faster TTS.

Supplementary Note 11: Real-valued gradient computing hardware.

Fig. S22 shows a possible detailed circuit implementation of the real-valued gradient computing hardware outlined in Fig. S21b. The forward and backward arrays comprise of crosspoint devices with linear and exponential input-to-output characteristics respectively. Memristors operating in their linear regime (with read voltage < 0.2V) and floating-gate memory cells operating in their subthreshold regime are chosen for the forward and backward crosspoint devices respectively, for this specific case. Using floating-gate memory or any other device with exponential trend in their input-to-output characteristics, enables performing the exponent operation locally at each crosspoint device. Devices with linear trends would necessitate addition of an anti-log amplifier at the input side of the backward array. For the forward array, memristors are chosen for their rather simpler (enabled by ohm's law) resistive behavior. However, it is noted that with some peripheral tweaks, even floating-gate cells can be used as linear crosspoint devices in the forward array (see Ref. 15). Note that the neuron blocks (in Fig. S22b) are only shown for completeness and not explicitly studied in this work. In an Ising machine with continuous-time dynamics, these blocks determine the update

mechanism/rule of the variables. One potential implementation of such a neuron block comprises of a leaky integrator followed by a nonlinear activation (see Ref. 16).

The variables are assumed to be within the range $[x_{\min}, x_{\max}]$, where $x_{\min} > 0$ and $x_{\max} \leq 1$. The analog voltages corresponding to the variables are denoted by x_i (where $i \in \{1,2,3,4\}$ in this example). For the version studied here, the mapping between the physical analog voltages depicting the polynomial's variables and their values in arbitrary units is 1V/a.u. Shifted version of these voltages given by x_i' $(= x_i + v_{\mathrm{b1}})$, undergo a log transformation, using an opamp with a diode connected between its output and inverting input to generate the internal voltages V_i'' (see equation (54), where m_1 is the product of thermal voltage and the subthreshold factor of the diode). This is then followed by an amplification stage with gain ($-A_{\mathrm{V1}}$) to generate voltages V_{xi} (see equation (55)) that are then applied to the word lines of the forward crossbar array. The gain A_{V1} is required to amplify the log compressed into the dynamic range of the memristor input read voltage.

$$V_i'' = v_{\mathrm{b1}} - m_1 \ln \frac{x_i' - v_{\mathrm{b1}}}{R_1 I_{\mathrm{S}}} \tag{54}$$

$$V_i = v_{\mathrm{b1}} + A_{\mathrm{V1}} m_1 \ln \frac{x_i' - v_{\mathrm{b1}}}{R_1 I_{\mathrm{S}}} \tag{55}$$

The bit line voltage of the forward array is equal to v_{bot} (see equation (56)) and is set by the bias voltage applied to the non-inverting end of the TIAs.

$$V_{\mathrm{bot}} = v_{\mathrm{b1}} - A_{\mathrm{V1}} m_1 \ln R_1 I_{\mathrm{S}} \tag{56}$$

The size of the forward array is N x (M+1), where N and M denote the number of variables and monomials in the polynomial respectively. The extra bit line is to compute an offset current that is subtracted from the other bit line currents (see equation (57)).

$$V_{\mathrm{off}}^{\mathrm{TIA}} = v_{\mathrm{bot}} - R_{\mathrm{f}} G_{\mathrm{off}} A_{\mathrm{V1}} m_1 \ln \prod_{i=1}^{N} (x_i' - v_{\mathrm{b1}}) \tag{57}$$

This offset current captures the leakage currents flowing through OFF state memristors with activated word lines in each bit line. Note that such an offset correction could potentially be used in the gradient computing hardware for binary valued variables but is not necessary due to wider margins. On the other hand, in the real-valued gradient computing hardware, currents in the forward array are log compressed and vary continuously in their valid range, resulting in narrower margins. The TIAs at the output of the forward crossbar array convert the bit line currents to proportional voltages (see equation (58), where t_j denote the jth monomial and E the set of all edges connecting monomials with their member variables) that are subtracted from the voltage of the offset bit line's TIA output using difference amplifiers. The difference amplifier outputs constitute control gate voltages for the floating-gate cells in the backward array and are proportional to the log of monomial products (see equation (59)).

$$V_j^{\mathrm{TIA}} = v_{\mathrm{bot}} - R_{\mathrm{f}} G_{\mathrm{on}} A_{\mathrm{V1}} m_1 \ln \prod_{i \,\forall \{x_i, t_j\} \in E} (x_i' - v_{\mathrm{b1}}) - R_{\mathrm{f}} G_{\mathrm{off}} A_{\mathrm{V1}} m_1 \ln \prod_{i \,\forall \{x_i, t_j\} \notin E} (x_i' - v_{\mathrm{b1}}) \tag{58}$$

$$V_{cj} = V_{\mathrm{off}}^{\mathrm{TIA}} - V_j^{\mathrm{TIA}} = R_{\mathrm{f}} (G_{\mathrm{on}} - G_{\mathrm{off}}) A_{\mathrm{V1}} m_1 \ln \prod_{i \,\forall \{x_i, t_j\} \in E} (x_i' - v_{\mathrm{b1}}) \tag{59}$$

The source line voltage of the floating-gate cells in the backward array denoted by V_{sl}, are given by expression in equation (60) (generated by passing the internal voltages V_l'' through an amplification stage with gain A_{V2} and bias voltage v_{b2}).

$$V_{sl} = (1 + A_{\mathrm{V2}}) v_{\mathrm{b2}} - A_{\mathrm{V2}} v_{\mathrm{b1}} + A_{\mathrm{V2}} m_1 \ln \frac{x_l' - v_{\mathrm{b1}}}{R_1 I_{\mathrm{S}}} \tag{60}$$

On setting $A_{\mathrm{V2}} = R_{\mathrm{f}} (G_{\mathrm{on}} - G_{\mathrm{off}}) A_{\mathrm{V1}}$, the net control-gate to source voltage drop across the floating-gate cells and the subthreshold current through them are given by equation (61) and (62) respectively. Here $V_{j,l}^{\mathrm{th}}$ is the threshold voltage of the cell sharing the jth control-gate and l^{th} source line and m_2 is the product of subthreshold factor and thermal voltage of the device.

$$V_{j,l}^{\text{cgs}} = V_{cj} - V_{sl} = R_{\text{f}}(G_{\text{on}} - G_{\text{off}})A_{\text{V1}}m_1 \ln \frac{\prod_{i\,\forall\,\{x_i,t_j\}\in E}(x_i' - v_{\text{b1}})}{x_l' - v_{\text{b1}}} - (1 + A_{\text{V2}})v_{\text{b2}} + A_{\text{V2}}v_{\text{b1}} + A_{\text{V2}}m_1 \ln R_1 I_{\text{S}} \quad (61)$$

$$I_{j,l} = I_0 e^{\frac{V_{j,l}^{\text{cgs}} - V_{j,l}^{\text{th}}}{m_2}} \quad (62)$$

The threshold voltage of the cells is set proportional to the coefficient (a_j) of the jth monomial and is given by equation (63), where V_{t0} is the threshold of the cells that correspond to monomials with coefficient equal to one. Monomials with higher coefficient values (a_j>1) have lower threshold voltages, resulting in higher subthreshold currents.

$$V_{j,l}^{\text{th}} = V_{\text{t0}} - m_2 \ln a_j \quad (63)$$

On setting $R_{\text{f}}(G_{\text{on}} - G_{\text{off}})A_{\text{V1}}m_1 = m_2$, substituting equation (60), (62) & (63) in (61) and rearranging the terms, one obtains an expression for the current given by equation (64), where the constant I_0'' is given by the expression in equation (65).

$$I_{j,l} = I_0 e^{\frac{m_2 \ln \frac{\prod_{i\,\forall\,\{x_i,t_j\}\in E}(x_i' - v_{\text{b1}})}{x_l' - v_{\text{b1}}} - \left(1 + {}^{m_2}/_{m_1}\right)v_{\text{b2}} + {}^{m_2}/_{m_1}\, v_{\text{b1}} + m_2 \ln R_1 I_{\text{S}} - V_{\text{t0}} + m_2 \ln a_j}{m_2}} = I_0'' \frac{a_j \prod_{i\,\forall\,\{x_i,t_j\}\in E}(x_i' - v_{\text{b1}})}{x_l' - v_{\text{b1}}} =$$
$$I_0'' \frac{a_j \prod_{i\,\forall\,\{x_i,t_j\}\in E} x_i}{x_l} \quad (64)$$

$$I_0'' = I_0 e^{\frac{-\left(1 + {}^{m_2}/_{m_1}\right)v_{\text{b2}} + {}^{m_2}/_{m_1}\, v_{\text{b1}} + m_2 \ln R_1 I_{\text{S}} - V_{\text{t0}}}{m_2}} \quad (65)$$

$$\frac{\partial H}{\partial x_l} \propto I_l = \sum_{j=1}^{M} I_{j,l} = I_0'' \sum_{j=1}^{M} \frac{a_j \prod_{i\,\forall\,\{x_i,t_j\}\in E} x_i}{x_l} \quad (66)$$

The expression of the current in equation (64) is proportional to the partial-derivative of the jth monomial with respect to variable x_l (provided they are in the range $[x_{\text{min}}, x_{\text{max}}]V$). The currents from all the cells in a column get summed up (according to Kirchoff's law) and this sum is proportional to the partial derivative of the high-degree polynomial function with respect to the variable corresponding to that column (see equation (66)). It is worth noting that while the cost/energy function of most combinatorial optimization are defined with variables $x \in [0,1]$, a new energy function can be defined with variables $x_{\text{new}} \in [x_{\text{min}}, x_{\text{max}}]$, by substituting x with $(x_{\text{new}} - x_{\text{min}})/(x_{\text{max}} - x_{\text{min}})$ in the original function. Furthermore, while the current hardware description is only valid for positive monomial coefficients ($a_j \geq 0$), a differential mode implementation with two-bit lines per variable in the backward array can enable both positive and negative monomial coefficients.

Each threshold voltage that is programmed to each floating gate cell has the following range: $V^{\text{th,min}} \leq V_{j,l}^{\text{th}} \leq V_{\text{t0}} < V^{\text{th,max}}$. The threshold voltage $V_{j,l}^{\text{th}} = V_{\text{t0}}$ when $a_j = 1$ (see equation (63)). The absolute maximum value ($V^{\text{th,max}}$) is determined by the device properties and $V_{j,l}^{\text{th}} = V^{\text{th,max}}$ if $\{x_i, t_j\} \notin E$ (or in other words, variable x_i is not present in monomial t_j). Equation (67) describes the relationship between the coefficient bit-precision (δ, in number of bits) and the threshold voltage limits when tuning error is assumed to be zero. The effective bit-precision considering non-zero threshold tuning error is lesser than this value for the same threshold voltage range.

$$\delta = \frac{V_{\text{t0}} - V^{\text{th,min}}}{m_2 \ln 2} \quad (67)$$

To maintain subthreshold operation regime, the control-gate to source voltage applied across each floating-gate cell is limited by $0 < V_{j,l}^{\text{cgs}} < V^{\text{th,min}}$. According to equation (61), the maximum limit is achieved for the case shown in equation (68) (when the corresponding monomial comprises of two variables, with both equal to the maximum value, x_{max}).

$$V_{j,l}^{\text{cgs}} = m_2 \ln x_{\text{max}} - \left(1 + {}^{m_2}\!/_{m_1}\right) v_{\text{b2}} + {}^{m_2}\!/_{m_1}\; v_{\text{b1}} + \; m_2 \ln R_1 I_{\text{S}} = V^{\text{th,min}} \tag{68}$$

On rearranging the terms in equation (68), it gives an expression for a minimum value for the bias voltage v_{b2}, as described in equation (69). The bias voltage is ultimately set to this value to allow maximum

$$v_{\text{b2}} \geq \frac{m_2 \ln x_{\text{max}} + {}^{m_2}\!/_{m_1}\, v_{\text{b1}} + m_2 \ln R_1 I_{\text{S}} - V^{\text{th,min}}}{1 + {}^{m_2}\!/_{m_1}} \tag{69}$$

The minimum limit for $V_{j,l}^{\text{cgs}}$ is reached when all the variables in the monomial with the highest degree (*K*) are set to the minimum value, x_{min} (see equation (70)). Substituting for the minimum value for the bias voltage v_{b2} and rearranging the terms, one gets the expression in equation (71).

$$V_{j,l}^{\text{cgs}} = m_2 (K-1) \ln x_{\text{min}} - \left(1 + {}^{m_2}\!/_{m_1}\right) v_{\text{b2}} + {}^{m_2}\!/_{m_1}\; v_{\text{b1}} + \; m_2 \ln R_1 I_{\text{S}} = 0 \tag{70}$$

$$K \leq 1 + \frac{\ln x_{\text{max}} - {}^{V^{\text{th,min}}}\!/_{m_2}}{\ln x_{\text{min}}} \tag{71}$$

For a given range for the variables $(x_{\text{min}}, x_{\text{max}})$, this highlights a trade-off in the maximum order of the monomial with the bit-precision of the monomial coefficients. Note that for $x_{\text{max}} \leq 1$, the denominator in equation (71) is negative and therefore for larger values of the maximum degree K of the polynomial, higher values of $V^{\text{th,min}}$ is required. Higher values for $V^{\text{th,min}}$ mean lesser bit-precision for monomial coefficients. Furthermore, for a fixed value of $V^{\text{th,min}}$, there exists a trade off between the maximum order and the dynamic range of the variables ($\zeta = x_{\text{max}}/x_{\text{min}}$). Larger values of *K* are possible with smaller values of *ζ*, which also results in smaller swing in the forward crossbar array input voltages, V_{xi}. Therefore, for smaller values of *ζ*, higher values of the gain $A_{\text{V1}}$is required. This results in higher power consumption and susceptibility to PVT variations.

Fig. S23 shows simulation results for real-valued partial-derivatives with respect to each variable in the polynomial described in Fig. S22a. The abscissa shows the ideal partial-derivative values whereas the left and right ordinates show the measured partial-derivative and the relative percent error respectively. All parameter values used in the simulations are detailed in Table S6. The simulations were done based on behavioral modeling of all hardware components described in Fig. S22b except for the neuron blocks. Error in the gradient computation is attributed to the modeled tuning errors of the memristors and floating-gate cells used in the forward and backward arrays respectively. Potential future work would be to study the impact of real-valued gradient computation error on the performance of continuous-time high order Hopfield neural networks by including appropriate models for the neuron activation blocks.

Supplementary Note 12: Towards Multi-Tile Architectures.

Although SAT problems of up to 100 variables have been shown to be efficiently solved by the proposed hardware, many high-order optimization problems of industrial and scientific relevance have thousands of variables. These problems cannot be mapped entirely to a single crossbar, as array dimensions are limited by IR drop issues. One solution to this problem as observed in the neuromorphic accelerator design community is to naively break up a larger "logical" crossbar into multiple smaller physical crossbar circuits (see Ref. 17-18). For example, in the ISAAC architecture[17], a tile hosting a limited-size physical memory array generates analog partial dot products. Partial product contributions from different tiles are summed in the digital domain after they undergo an Analog-to-Digital Conversion (ADC). Such a technique could work for dense large-scale high-order optimization problems.

However, the PUBO and CNF of most practical optimization problems are sparse, with the sparsity increasing with problem size[19]. They also have limited fan-in (number of monomials/clauses a variable is a part of) with the maximum fan in being several orders smaller than the actual number of variables. As a result, our analysis shows that when a sparsity and fan-in aware embedding is done, all nontrivial weights (G_{on}) of a variable/literal can be packed in a single physical crossbar (without exceeding critical linear dimension limits enforced by IR drop issues) with the dot-product being computed entirely in the analog domain and avoiding expensive ADCs. Such a sparsity-aware architecture was recently proposed for implementing second-order Ising Machines (see Ref. 19).

However, such an architecture can only capture second-order interactions and is therefore limited to solving QUBOs. Figure. S24 shows how such architectures can be extended to compute high-degree gradients and implement high-

order Ising machines in a multi-tiled fashion. Such an architecture is inspired from Field Programmable Gate Arrays (FPGAs) (see Ref. 20), where the cluster-based logic blocks are replaced by In-Memory Computing (IMC) cores that are present as *islands* in a *sea* of programmable routing fabric (see Fig. S24a). The routing fabric comprises of interconnects, switches and connection blocks that connect the inputs and outputs of the IMC cores. The programming circuit required to tune the memristors is shared by all tiles and is present in the periphery. Figure. S24b shows the detailed view of a single tile. The IMC core comprises of two crossbar arrays: the forward and the backward arrays, and their associated peripheral circuits. The forward array receives its variable/literal inputs both locally (from the variable/literal register array associated with the backward array of the same IMC core) and globally via the connection blocks (arising from the variable/literal register arrays associated present in other IMC cores and communicated using the routing fabric). In a similar fashion, the backward array receives its clause/monomial inputs both locally and globally. The outputs of both arrays in each IMC core are communicated to the routing fabric via the connection blocks. Since all the inputs and outputs of the forward and backward arrays are not connected to each other, the dimensions of the arrays; (x_1, y_1) and (x_2, y_2) respectively, are not the same. These dimensions are determined by problem specific parameters like maximum fan-in and sparsity, as well as the specific packing algorithm used.

To demonstrate such packing, we leverage an open-source place and route tool called Versatile Place and Route (VPR), developed for FPGA design (see Ref. 21) to embed high-degree Boolean satisfiability problems to the proposed architecture. In the packing step, the goal is to simultaneously pack as many clauses (variables/literals) sharing the same variable/literal (clause) inputs in the forward (backward) arrays of each IMC core, to minimize the number of signals that need to be routed between the cores as well as to attempt to fill each crossbar array to its capacity, to minimize the total number of cores required. As a result of this optimization, a large number of trivial weights (memristors with conductance equal to G_{off}) are filtered out. This results in the total area of all crossbars spread out among the multiple IMC cores being much less than the area of the single "logical" crossbar representing the high-order optimization problem before packing. Furthermore, each variable/literal/clause ends up having all their non-trivial inputs connections (fan-ins) in the same array. This allows parallel gradient computation without requiring ADCs for sparse problems. Figure S24c shows the compression in the crossbar areas achieved because of such packing for the problems considered in Fig. S11.

Supplementary References

1. Jordán, C. Calculus of Finite Differences. *American Mathematical Soc*. (1965).
2. Sheng, Xia, *et al.* Low-conductance and multilevel CMOS-integrated nanoscale oxide memristors. *Advanced Electronic Materials* **5.9** 1800876 (2019).
3. Mu, Chen, *et al*. A 200M-Query-Vector/s Computing-in-RRAM ADC-less k-Nearest-Neighbor Accelerator with Time-Domain Winner-Takes-All Circuits. *2022 IEEE 4th International Conference on Artificial Intelligence Circuits and Systems (AICAS)* 222-225 (2022).
4. Cai, F. *et al.* Power-efficient combinatorial optimization using intrinsic noise in memristor Hopfield neural networks. *Nature Electronics* **3** 409-418 (2020).
5. Kanazawa, K. and Maruyama, T. An approach for solving large SAT problems on FPGA. *ACM Transactions on Reconfigurable Technology and Systems (TRETS)* ***4*.1** 1-21 (2010).
6. Hoos, H. *SATLIB — Benchmark problems* (2011) Available online at https://www.cs.ubc.ca/~hoos/SATLIB/benchm.html
7. Inagaki, Takahiro, *et al*. A coherent Ising machine for 2000-node optimization problems. *Science* **354.6312** 603-606 (2016).
8. Willsch, Dennis, *et al*. Benchmarking Advantage and D-Wave 2000Q quantum annealers with exact cover problems. *Quantum Information Processing* **21.4** 141 (2022).
9. Aadit, Navid Anjum, *et al*. Massively parallel probabilistic computing with sparse Ising machines. *Nature Electronics* **5.7** 460-468 (2022).
10. Sharma, A. *et al.* Augmented electronic Ising machine as an effective SAT solver. *Nature Scientific Reports* **13** 22858 (2023).
11. Reifenstein, Sam, *et al.* Coherent SAT solvers: a tutorial. *Advances in Optics and Photonics* **15.2** 385-441 (2023).
12. https://www.xilinx.com/publications/technology/power-advantage/7-series-power-benchmark-summary.pdf
13. https://www.nextplatform.com/2018/10/01/boosting-the-clock-for-high-performance-fpga-inference/
14. Hizzani, M. *et al.* Memristor-based hardware and algorithms for higher-order Hopfield optimization solver outperforming quadratic Ising machines. ArXiv:2311.01171 (2023).
15. Guo, Xinjie, *et al*. Temperature-insensitive analog vector-by-matrix multiplier based on 55 nm NOR flash memory cells. *2017 IEEE Custom Integrated Circuits Conference (CICC)* 1-4 (2017).
16. Joya, G., Atencia, M.A. & Sandoval, F. Hopfield neural networks for optimization: Study of the different dynamics. *Neurocomputing* **43** 219-237 (2002).
17. A. Shafiee, *et al*. ISAAC: A convolutional neural network accelerator with in-situ analog arithmetic in crossbars *ACM SIGARCH Computer Architecture News* **44.3** 14-26 (2016).

18. Bavandpour, M., Mahmoodi, M.R. & Strukov, D.B. aCortex: An energy-efficient multi-purpose mixed-signal inference accelerator. *IEEE Journal on Exploratory Solid-State Computational Devices and Circuits* **6** 98-106 (2020).
19. Hutchinson, George Higgins, *et al*. FPIA: Field-Programmable Ising Arrays with In-Memory Computing. *arXiv preprint arXiv* ***2401.16202*** (2024).
20. V. Betz, J. Rose, and A. Marquardt. Architecture and CAD for deep submicron FPGAs. *Springer Science & Business Media* **497** (2012).
21. V. Betz and J. Rose. VPR: A new packing, placement and routing tool for FPGA research. *Lecture Notes in Computer Science* **1304** 213–222 (1997).
22. Li, C. *et al.* CMOS integrated nanoscale memristive crossbars for CNN and optimization acceleration. *IMW'20* (2020).
